# Target oxidative stress-induced disulfidptosis: novel therapeutic avenues in Parkinson’s disease

**DOI:** 10.1186/s13041-025-01200-2

**Published:** 2025-04-04

**Authors:** Junshi Zhang, Tingting Liu, Haojie Wu, Jianshe Wei, Qiumin Qu

**Affiliations:** 1 Department of Neurology, The First Affiliated Hospital of Xi’an Jiaotong University, Xi’an, 710061 China; 2 Department of Neurology, Huaihe Hospital of Henan Universtiy, Kaifeng, 475004 China; 3 Institute for Brain Sciences Research, School of Life Sciences, Henan University, Kaifeng, 475004 China

**Keywords:** Parkinson’s disease, Oxidative stress, Disulfidptosis, Mitochondria

## Abstract

**Background:**

Parkinson’s disease (PD), a globally prevalent neurodegenerative disorder, has been implicated with oxidative stress (OS) as a central pathomechanism. Excessive reactive oxygen species (ROS) trigger neuronal damage and may induce disulfidptosis—a novel cell death modality not yet characterized in PD pathogenesis.

**Method:**

Integrated bioinformatics analyses were conducted using GEO datasets to identify PD-associated differentially expressed genes (DEGs). These datasets were subjected to: immune infiltration analysis, gene set enrichment analysis (GSEA), weighted gene co-expression network analysis (WGCNA), intersection analysis of oxidative stress-related genes (ORGs) and disulfidptosis-related genes (DRGs) for functional enrichment annotation. Following hub gene identification, diagnostic performance was validated using independent cohorts. LASSO regression was applied for feature selection, with subsequent experimental validation in MPTP-induced PD mouse models. Single-cell transcriptomic profiling and molecular docking studies were performed to map target gene expression and assess drug-target interactions.

**Result:**

A total of 1615 PD DEGs and 200 WGCNA DEGs were obtained, and the intersection with ORGs and DRGs resulted in 202 DEORGs, 11 DEDRGs, and 5 DED-ORGs (NDUFS2, LRPPRC, NDUFS1, GLUD1, and MYH6). These genes are mainly associated with oxidative stress, the respiratory electron transport chain, the ATP metabolic process, oxidative phosphorylation, mitochondrial respiration, and the TCA cycle. 10 hub genes have good diagnostic value, including in the validation dataset (AUC ≥ 0.507). LASSO analysis of hub genes yielded a total of 6 target genes, ACO2, CYCS, HSPA9, SNCA, SDHA, and VDAC1. In the MPTP-induced PD mice model, the expression of ACO2, HSPA9, and SDHA was decreased while the expression of CYCS, SNCA, and VDAC1 was increased, and the expression of the 5 DED-ORGs was decreased. Additionally, it was discovered that N-Acetylcysteine (NAC) could inhibit the occurrence of disulfidptosis in the MPTP-induced PD model. Subsequently, the distribution of target genes with AUC > 0.7 in different cell types of the brain was analyzed. Finally, molecular docking was performed between the anti-PD drugs entering clinical phase IV and the target genes. LRPPRC has low binding energy and strong affinity with duloxetine and donepezil, with binding energies of -7.6 kcal/mol and − 8.7 kcal/mol, respectively.

**Conclusion:**

This study elucidates the pathogenic role of OS-induced disulfidptosis in PD progression. By identifying novel diagnostic biomarkers (e.g., DED-ORGs) and therapeutic targets (e.g., LRPPRC), our findings provide a mechanistic framework for PD management and lay the groundwork for future therapeutic development.

**Supplementary Information:**

The online version contains supplementary material available at 10.1186/s13041-025-01200-2.

## Highlight

Uncovers the key role of oxidative stress in PD progression through disulfidptosis mechanism. Identifies 5 novel DED-ORGs (NDUFS2, LRPPRC, NDUFS1, GLUD1, MYH6) involved in both oxidative stress and disulfidptosis. Validates 10 hub genes with diagnostic potential and 6 target genes in MPTP-induced PD mice model. Reveals cell-type-specific expression of target genes and evaluates binding affinities of anti-PD drugs with the identified targets.

Parkinson’s disease (PD), ranked as the second most prevalent neurodegenerative disorder worldwide, currently affects over 6 million individuals globally. This figure reflects a 2.5-fold increase in prevalence over the past generation, resulting in its recognition as one of the leading causes of neurological disability worldwide [1]. The neuropathological hallmark of PD manifests as neuronal inclusions termed Lewy bodies (LBs) and Lewy neurites, concomitant with progressive neuronal loss in both the substantia nigra (SN) and multiple extra-nigral brain regions [2]. While the pathogenesis of PD remains incompletely understood, emerging evidence suggests the involvement of multiple interacting pathophysiological mechanisms, including but not limited to oxidative stress (OS), neuroinflammation, mitochondrial dysfunction, pathological protein aggregation and propagation, autophagic flux impairment, dysregulation of apoptotic pathways, and gut microbiome dysbiosis [3]. Notably, accumulating evidence positions OS as a central driver of the degenerative cascade underlying dopaminergic neurodegeneration across all forms of PD [4].

Oxidative stress (OS) arises from dysregulated cellular redox homeostasis, characterized by an imbalance between reactive oxygen species (ROS) production and their clearance via endogenous antioxidant systems comprising enzymes and molecular chaperones. Critically, OS per se is not inherently pathological; instead, neuronal damage is mediated by ROS accumulation resulting from sustained redox imbalance [5]. Emerging evidence from early-stage PD patients demonstrates that elevated OS represents a hallmark feature during disease initiation, preceding substantial neuronal degeneration. These findings imply that uncontrolled ROS generation may act as a primary instigator of dopaminergic neuron death, rather than constituting a mere secondary consequence of neurodegeneration [6, 7]. Elucidating the multifaceted role of OS in PD pathogenesis could thus provide novel therapeutic targets and biomarkers for preclinical diagnosis.

Disulfidptosis, a recently discovered cell death modality, differs fundamentally from canonical programmed cell death pathways such as apoptosis, necroptosis, pyroptosis, autophagy, ferroptosis, or cuproptosis [8]. This process is defined by rapid progression initiated through intracellular cysteine overload induced by disulfide stress. Such metabolic perturbation promotes aberrant disulfide bonding within actin cytoskeletal proteins, thereby disrupting actin filament networks and precipitating catastrophic cell collapse. Given that disulfide generation is intrinsically linked to OS, deciphering the mechanistic relationship between disulfide accumulation and cell death holds significant implications. This novel cell death paradigm was co-discovered through collaborative efforts between Professor Ganapathy-Kanniappan’s team at MD Anderson Cancer Center (USA) and Professor Chen Junjie’s research group (China) [9]. Under nutrient-deprived conditions (e.g., glucose starvation) combined with impaired repair mechanisms, cells exhibiting high solute carrier family 7 member 11 (SLC7A11) expression develop pathological disulfide accumulation. This triggers disulfide stress culminating in disulfidptosis—a mechanistically distinct cell death form with unique cytoskeletal destabilization features. Specifically, excessive disulfide bonds within actin filaments promote cytoskeletal contraction and structural disintegration, irreversibly compromising cellular integrity. Targeted inhibition of specific cell death pathways has shown therapeutic efficacy in neurodegenerative diseases [10, 11], suggesting that disulfidptosis modulation may offer innovative treatment strategies for PD and related disorders.

Emerging evidence highlights a complex interplay between OS and disulfidptosis in the pathogenesis of neurodegenerative diseases. Systematic identification of key biomarkers could uncover novel therapeutic avenues for these age-associated disorders. In this multi-omics investigation, we first retrieved PD-related differentially expressed genes (DEGs) from the GEO database (Accession: https://www.ncbi.nlm.nih.gov/geo/) [12], which were subsequently analyzed for immune microenvironment profiling and pathway enrichment via Gene Set Enrichment Analysis (GSEA). Following this, a tripartite integration strategy was employed to intersect PD-DEGs with disulfidptosis-related genes (DRGs) and OS-associated genes (ORGs), enabling functional enrichment analysis and hub gene prioritization. The translational potential of these hub genes and differentially expressed disulfidptosis-OS-related genes (DED-ORGs) was rigorously validated through diagnostic performance evaluation and mechanistic interrogation. Furthermore, computational mapping of hub gene-drug interaction sites revealed druggable targets for clinically approved anti-PD agents. Collectively, this framework provides a molecular blueprint for rational drug design and personalized therapeutic strategies, while elucidating the synergistic roles of OS and disulfidptosis in neurodegeneration. These advances are poised to redefine mechanistic understanding in PD research, accelerate biomarker-driven clinical trials, and ultimately enhance patient outcomes through precision medicine approaches.

## Materials and methods

### Data acquisition

**Fig. 1:**
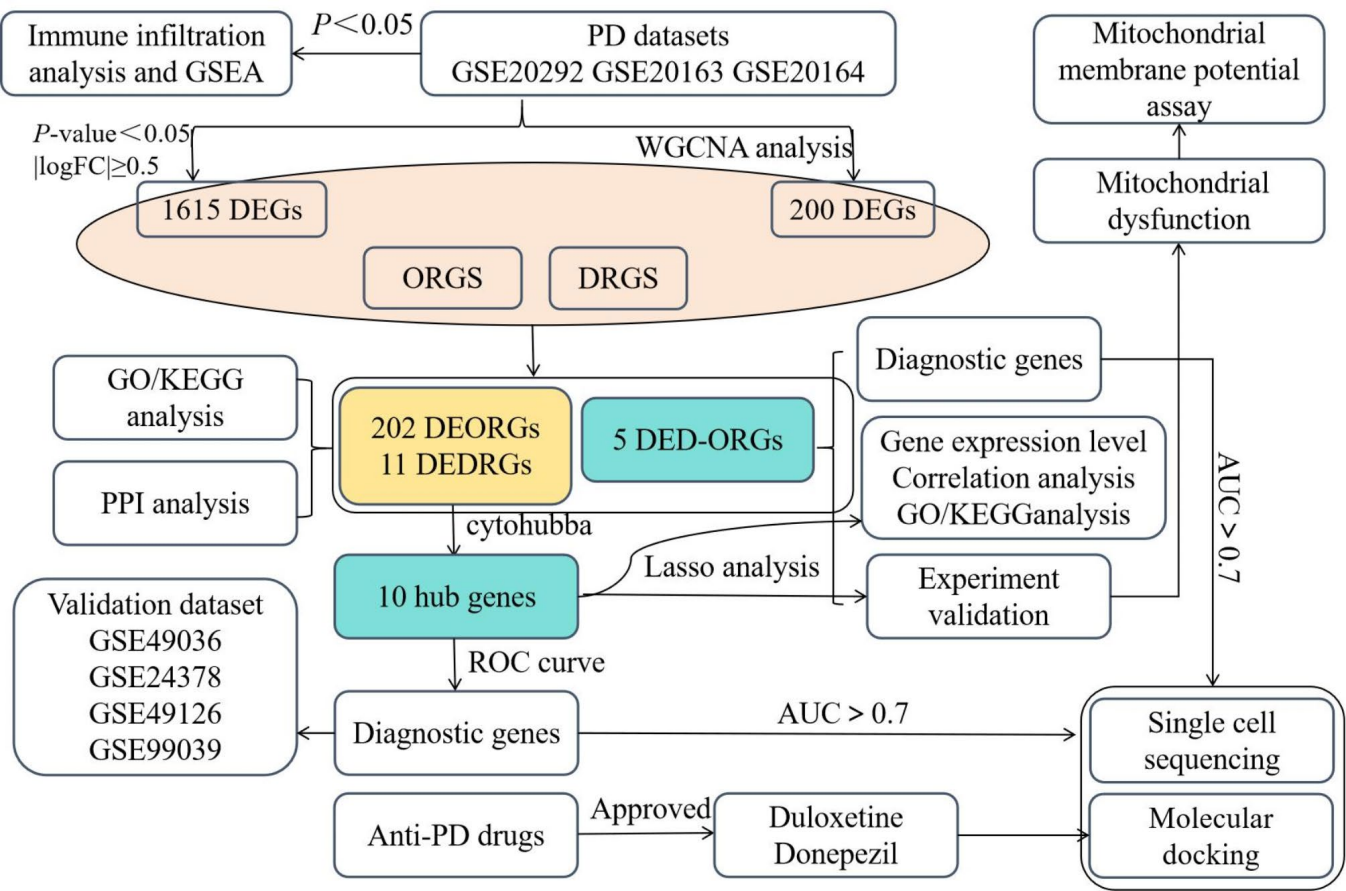
The flow chart of this study. GSEA, gene set enrichment analysis; WGCNA, weighted gene co-expression network analysis; DEGs, diferentially expressed genes; ORGs, oxidative stress-related genes; DRGs, disulfidptosis-related genes; DEORGs, diferentially expressed oxidative stress-related genes; DEDRGs, diferentially expressed disulfidptosis-related genes; DED-ORGs, diferentially expressed disulfidptosis-oxidative stress-related genes; LASSO, Least Absolute Shrinkage and Selection Operator; GO, Gene Ontology; KEGG, Kyoto Encyclopedia of Genes and Genomes; ROC, receiver operating characteristic

In this study, the data analysis was completed using R software (version 4.2.1) and the following R packages: GEOquery (2.64.2) was used to download the datasets GSE20292, GSE20163, GSE20164, GSE49036, GSE24378, GSE49126, and GSE99039 from the GEO database; limma (3.52.2) was used for data normalization and differential analysis; and ggplot2 (3.4.4) and ComplexHeatmap (2.13.1) were responsible for visualization. The datasets information was listed in Table 1. Figure 1 depicts the flow chart of this study. 103 DRGs [13] were shown in Supplementary Table S1, and 1399 ORGs with the relevance score ≥ 7 were downloaded from the GeneCards database [14] (https://www.genecards.org) for further analysis (Supplementary Table S2).

**Table 1:** The detailed information of PD datasets

GEO dataset	Platform	Sample information
GSE20292	GPL96[HG-U133A] Affymetrix Human Genome U133A Array	11 PD patients and 18 healthy controls from SN samples
GSE20163		8 PD patients and 9 healthy controls from substantia nigra (SN) samples
GSE20164		6 PD patients and 5 healthy controls from SN samples
GSE49036	GPL570 [HG-U133_Plus_2] Affymetrix Human Genome U133 Plus 2.0 Array	Control Braak α-synuclein Stage 0: 8 samples; α Braak α-synuclein stage 1–2: 5 samples; Braak α-synuclein 3–4: 7 samples; Braak α-synuclein stages 5–6: 8 samples
GSE99039		233 healthy controls and 205 IPD patients from whole blood
GSE24378	GPL1352 [U133_X3P] Affymetrix Human X3P Array	human dopamine (DA) from postmortem brains of 8 PD patients and 9 healthy controls
GSE49126	GPL4133 Agilent−014850 Whole Human Genome Microarray 4 × 44 K G4112F (Feature Number version)	30 PD patients and 20 healthy controls from peripheral blood mononuclear cells (PBMC)

### GSEA and immune infiltration analysis

GSEA systematically evaluates the coordinated expression patterns of gene sets curated from the Molecular Signatures Database (MSigDB; https://www.gsea-msigdb.org/gsea/msigdb) [15] against phenotype-specific gene rankings. This computational approach determines the statistical association between predefined biological pathways and disease phenotypes. All analyses were performed using the clusterProfiler R package (v4.4.4) in R statistical environment (v4.2.1).Single-sample gene set variation analysis (ssGSEA) was implemented via the GSVA R package [16] to quantify immune cell abundance using transcriptomic data. A panel of 24 immune cell-type-specific gene signatures, derived from published immunological resources [17], was applied to compute infiltration scores reflecting tissue-level immune microenvironment composition. Comparative visualization of immune cell distributions between PD patients and healthy controls was achieved through boxplot analysis.

### Weighted gene co-expression network analysis (WGCNA)

WGCNA aimed to identify gene modules that are co-expressed and explore the association between gene networks and phenotypes of interest, as well as to identify hub genes within the networks. We established a weighted adjacency matrix, defined a correlation power (soft thresholding parameter) that demonstrated strong relations between genes and penalizing the weak correlation. Then we converted the adjacency into a topological overlap matrix (TOM) to measure the network connectivity of gene. The TOM summed the adjacent genes’ contributions to calculate the network gene ratio and determined. We used average linkage hierarchical clustering based on TOM dissimilarity measurements to classify genes with similar expression profiles into gene modules. These modules were represented by branches and different colors in the cluster tree. We constructed module relationships and screened genes accordingly [18].

### Differential expression genes analysis

Intersection analysis was visualized using the venneuler R package (v1.1.3) in R v4.2.0 to map overlapping relationships among DEGs, WGCNA DEGs, ORGs, and DRGs. The Kyoto Encyclopedia of Genes and Genomes (KEGG) pathway and Gene Ontology (GO) enrichment analysis of DEORGs, DEDRGs, and DED-ORGs were conducted using the “clusterProfiler” and “GOplot” packages in R software.Protein-protein interaction (PPI) networks were constructed through the STRING database (https://string-db.org) [19], with topological centrality evaluated by 12 Cytohubba algorithms in Cytoscape v3.9.0 to identify top 10 hub genes. Functional characterization of hub genes was annotated via the GeneCards database (https://www.genecards.org/). Additionally, the ggplot2 [3.3.6] and ggwordcloud [0.6.0] packages were used to visualize the frequency of hub gene occurrences. The hub genes were displayed through heatmaps and box plots in both PD patients and healthy controls. Spearman correlation analysis was conducted using R software version 4.2.1, specifically leveraging the ggplot2 [3.3.6] package for visualization, and the results were presented in a pie chart. Gene visualization in chromosome mapping was achieved using R software version 4.2.1 with the circularize [0.4.15] package.

### ROC and LASSO analysis

To identify diagnostic genes, we conducted receiver operating characteristic (ROC) curve visualization analysis and calculated the area under the curve (AUC) using the pROC package in R software, aiming to ascertain the predictive value of the hub gene. Genes with an AUC > 0.700 were selected as diagnostic genes. The Least Absolute Shrinkage and Selection Operator (LASSO) analysis, a widely adopted method for feature selection and regularization, is frequently utilized in both linear and logistic regression models. By employing R software (version 4.2.1) with the glmnet package (version 4.1.7), we analyzed the cleaned data to derive variable coefficients, logarithms of lambda values, L1 regularization parameters, lambda values, likelihood values, or classification error rates, and visualized the data accordingly. Additionally, we analyzed the expression of genes in various brain cells using the Human Transcriptome Cell Atlas database (https://www.htcatlas.org/).

### Drug prediction and molecular Docking

To predict PD-related therapeutic drugs, we utilized the Malacards database (https://www.malacards.org/). For analyzing the binding affinities and interaction modes between the drug candidates and their targets, we employed AutodockVina 1.2.2, a computer-aided protein-ligand docking software [20]. The molecular structures of the compounds were retrieved from PubChem Compound (https://pubchem.ncbi.nlm.nih.gov/) [21], while their 3D coordinates were downloaded from the Protein Data Bank (PDB) (http://www.rcsb.org/pdb/). Prior to docking analysis, all protein and molecular files were converted to the PDBQT format, with water molecules excluded and polar hydrogen atoms added. The grid box was centered to encompass the binding domain of each protein and to allow for unrestrained molecular movement. The dimensions of the grid box were set to 30 Å × 30 Å × 30 Å, with a grid point spacing of 0.34 Å (or 0.05 nm). The molecular docking studies were conducted using Autodock Vina 1.2.2 (http://autodock.scripps.edu/). Note: The grid point distance provided earlier was adjusted from 0.05 nm to 0.34 Å for accuracy, as 0.05 nm corresponds to 0.5 Å (which is not commonly used in docking studies), and 0.34 Å is a more typical value.

## In vivo and in vitro experimental verification

### Animal

Experimental protocols were conducted using male C57BL/6J mice (age: 6–8 weeks; weight: 25–30 g) procured from Cyagen Biosciences. F0-generation transgenic mice were crossed with wild-type counterparts at sexual maturity (8 weeks), yielding F1 progeny for experimental use. All animals were housed under controlled environmental conditions (12/12 h light/dark cycle; temperature: 22 ± 2 °C; humidity: 55 ± 5%) with ad libitum access to food and water. Animal procedures complied with the NIH Guide for the Care and Use of Laboratory Animals and were approved by the Institutional Animal Ethics Committee of Henan University (Approval No.:HUSOM2021-161). To minimize bias, experimenters were blinded to group assignments. Following a 14-day acclimatization period, mice were randomly allocated to four experimental groups (*n* = 8/group): Saline control (0.9% NaCl, i.p. ×45 days), MPTP-lesioned (1-methyl-4-phenyl-1,2,3,6-tetrahydropyridine; Abmole, Cat# M9049; 20 mg/kg/day, i.p. ×15 days) [22], NAC monotherapy (N-Acetylcysteine; MedChemExpress, Cat# HY-B0215; 1 g/kg/day, i.p. ×30 days), NAC + MPTP co-treatment. At study termination (Day 46), subsets of animals (*n* = 4/group) underwent transcardial perfusion with 0.9% saline followed by 4% paraformaldehyde (PFA) for histopathological analysis. Concurrently, fresh substantia nigra (SN) tissues were dissected under cryogenic conditions, flash-frozen in liquid nitrogen, and stored at − 80 °C for molecular analyses.

### Cell culture

The human dopaminergic neuroblastoma cell line SH-SY5Y (Servicebio, Wuhan, China) was maintained in high-glucose Dulbecco’s Modified Eagle Medium (DMEM; Gibco, Grand Island, NY) supplemented with 10% fetal bovine serum (FBS), 100 U/mL penicillin, and 100 µg/mL streptomycin. Cells were cultured at 37 °C in a 5% CO₂-humidified incubator, with medium replacement every 48–72 h. To establish in vitro Parkinson’s disease models, SH-SY5Y cells were exposed to 1 mM 1-methyl-4-phenylpyridinium (MPP⁺; Abmole, Cat# M10041) for 24 h [23], resulting in two experimental groups: control group and PD model group (MPP⁺-treated). For pharmacological intervention, cells pretreated with MPP⁺ were co-administered with either: Duloxetine (1 µM; MedChemExpress, Cat# HY-B0161) or Donepezil (1 µM; MedChemExpress, Cat# HY-14566). Following 4-hour drug exposure, cells were harvested for downstream analyses.

### Western blot

SN and SH-SY5Y cells was homogenized with a Polytron in ice-cold RIPA buffer supplemented with PMSF (catalog numbers G2002 and G2008, Servicebio, China). The samples were sonicated and cleared by centrifugation at 12,000×g for 10 min, at 4℃. Protein concentration in the supernatant was determined by BCA assay. The proteins were separated on SDS-PAGE gels and subsequently transferred onto a nitrocellulose membrane (Millipore, IPFL00010, Germany) by electrophoresis. The blots were blocked in 5% nonfat milk in TBST for 1 h at room temperature and then probed with primary antibody, including SLC7A11, ACTN4, MYL6, ACO2, CYCS, HSPA9, SNCA, SDHA, VDAC1, NDUFS2, LRPPRC, NDUFS1, GLUD1, MYH6, and β-actin (all from Affinity, Melbourne), diluted in TBST with 1% nonfat milk, overnight at 4℃. After overnight incubation, the blots were incubated with HRP-conjugated secondary antibody in TBST with 1% nonfat milk for 2 h at room temperature. Finally, the blots were developed using an Enhanced Chemiluminescence (ECL) assay (BIO-Rad).

### Immunohistochemistry assay

Immunohistochemistry was performed on 30-µm-thick serial brain sections. We used 1x Citrate Antigen Retrieval Solution at 98 °C for 10 min, followed by treatment with 0.1% Triton X-100 for 10 min to ensure transparency. The sections were then incubated with hydrogen peroxide for 20 min in the dark to block endogenous peroxidase activity. After that, they were blocked with 10% goat serum in PBS and incubated with tyrosine hydroxylase (TH) antibody (1:200, Affinity). The TH staining was detected using a DAB kit (catalog number: G1212, Servicebio). A microscope (Leica DMI4000B; Wetzlar, Germany) was used for observation. Densitometry analysis was performed on the scanned immunohistochemical images using ImageJ software.

### Mitochondrial membrane potential assay

Obtained the SN from the mice brain and used the Tissue Mitochondria Isolation Kit (Beyotime, C3606) to isolate mitochondria. Subsequently, the Mitochondrial Membrane Potential Assay Kit with JC-1 (Beyotime, C2006) to detect the mitochondrial membrane potential in the tissues and cells. Additionally, a ROS probe was utilized to detect the fluorescence intensity of ROS in the cells.

### Statistical analysis

In this study, R adaptation 4.2.0 software and Adobe Photoshop 2021 were utilized. The data were expressed as the mean ± SEM, and group comparisons were conducted using an unpaired Student’s t-test or One-Way ANOVA. The area under the ROC curve (AUC) and predictive accuracy were assessed. Statistical significance was defined as a *P*-value less than 0.05.

## Results

### DEGs acquisition of PD datasets

**Fig. 2:**
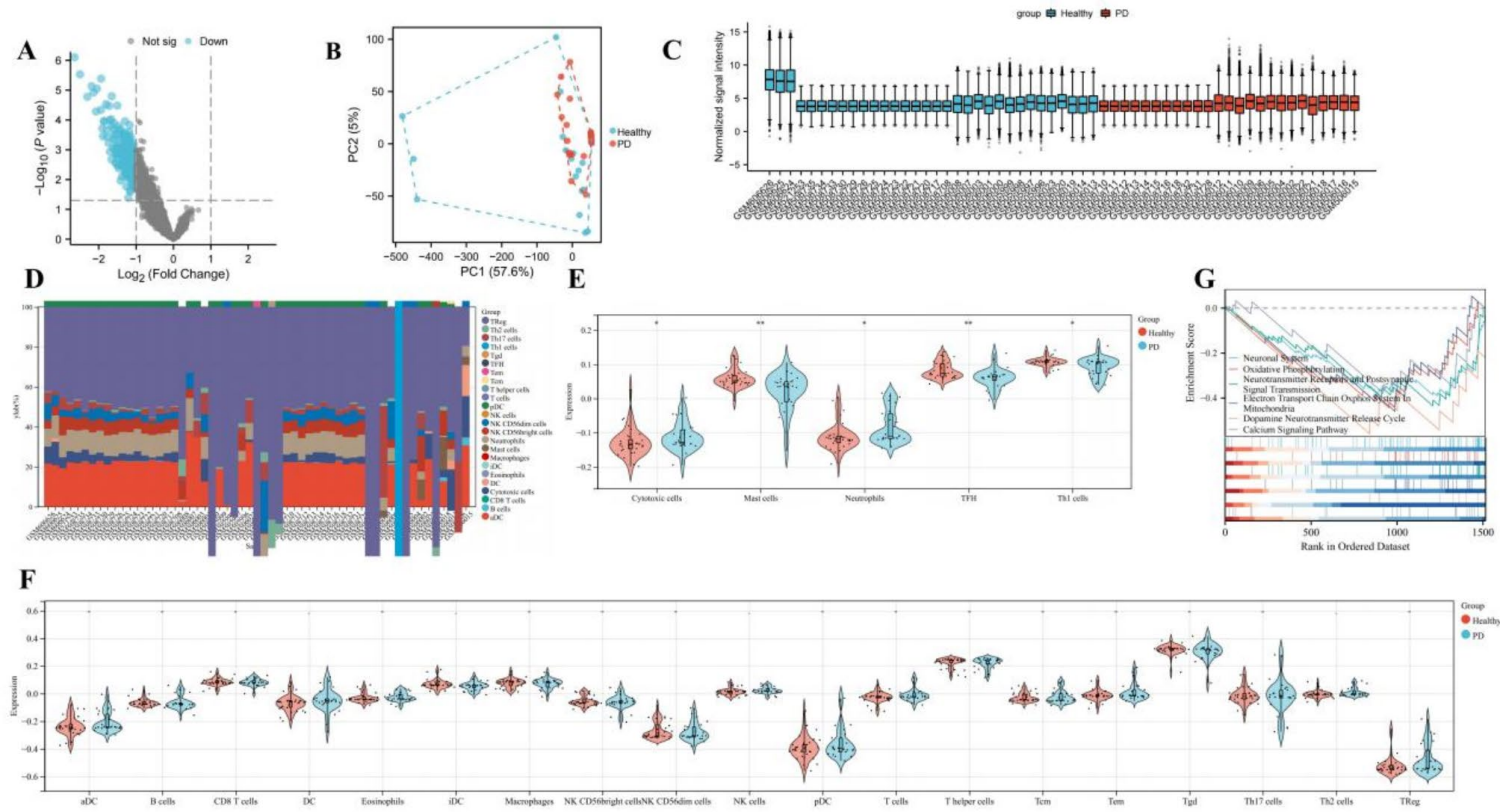
Gene chip information acquisition and analysis. (**A**) The differential gene volcano map. (**B**) PCA analysis. (**C**) The gene chip expression profiles of 57 brain tissue samples, including 25 PD patients and 32 healthy controls. (**D**) Boxplot of differentially expressed immune cells in the GSE20292, GSE20163, and GSE20164 datasets of PD. (**E**) and (**F**) Expression of immune cells in the PD group compared with the healthy controls. (**G**) GSEA enrichment analysis in the GSE20292, GSE20163, and GSE20164 datasets of PD. Compared with the healthy controls, *P < 0.05, **P < 0.01

Normalization was performed with samples from the GSE20163, GSE20164, and GSE20292 datasets, using thresholds of adjusted|logFC|>0.5 and *P*.adj<0.05. PCA and mean-variance trend analysis indicated that the data were reproducible and lacked significant trends, as observed in the distribution patterns of the box plots. Volcano plots were utilized to visualize the differential genes, resulting in the identification of 1615 DEGs in the PD datasets (Figs. 2A−2 C).

### GSEA and immune infiltration analysis

Heatmaps depicting the immune infiltration in the PD datasets are shown in Fig. 2D. Compared with healthy controls, Mast cells, TFH, and Th1 cells were lowly expressed, while Cytotoxic cells and Neutrophils were highly expressed (Fig. 2E and F). The GSEA showed that DEGs were mainly involved in the neuronal system, oxidative phosphorylation, neurotransmitter receptors and postsynaptic signal transmission, electron transport chain OXPHOS system in mitochondria, dopamine neurotransmitter release cycle, and calcium signaling pathway (Fig. 2G).

### WGCNA identification of DEGs

**Fig. 3:**
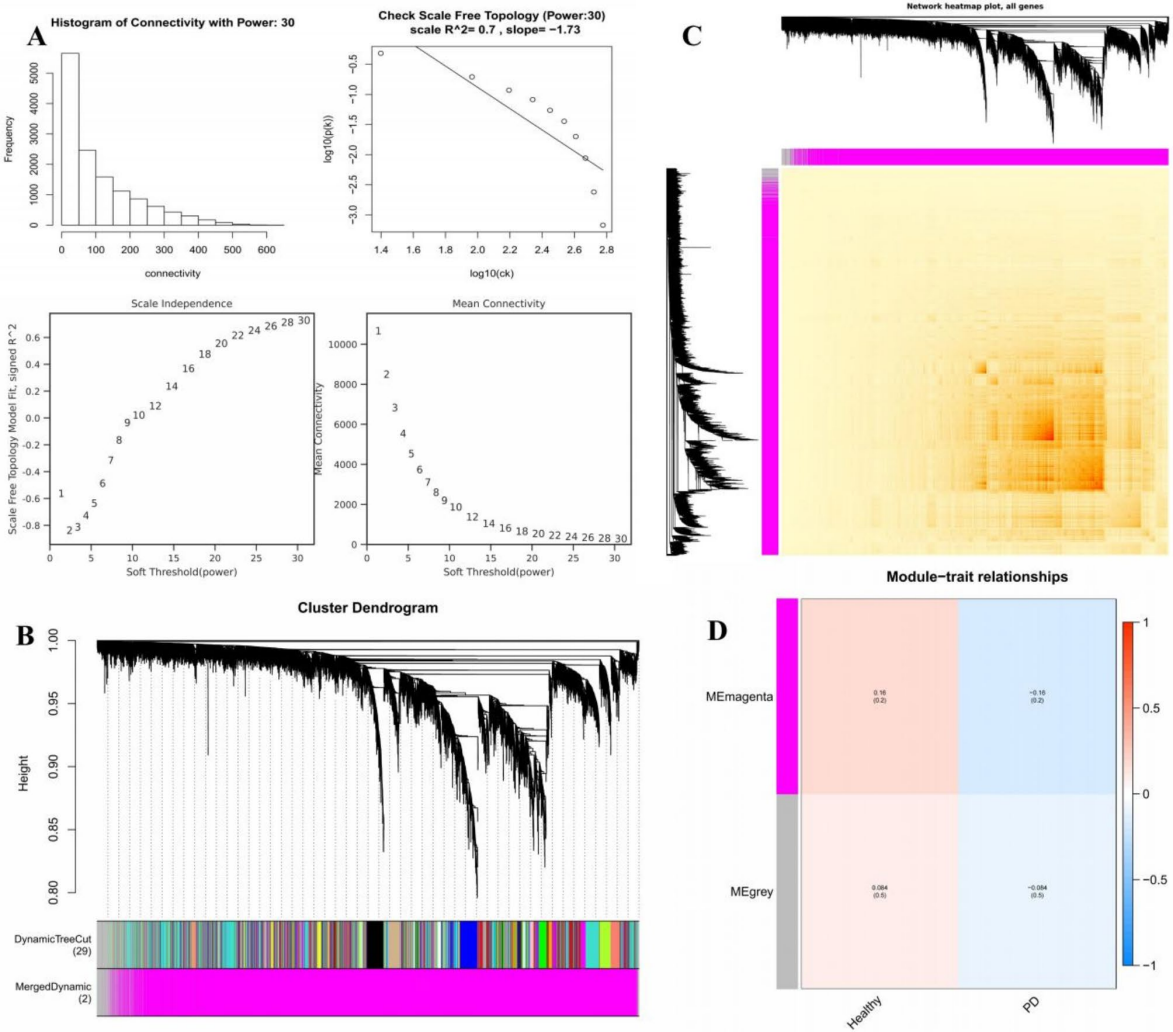
WGCNA analysis of the PD datasets. (**A**) WGCNA network construction parameters. (**B**) The upper middle part of the figure is the gene cluster tree constructed by the dissTOM matrix constructed by the weighted correlation coefficients. The color of Dynamic TreeCut is the module identified by the dynamicTreeCut method. Since there is a certain correlation between some modules, the corresponding modules are merged into the same module, that is, the merged dynamic below is the final module, and these modules are used for subsequent analysis. (**C**) Cluster heatmap of all genes. (**D**) Trait module association heatmap

Scale-free topology optimization required systematic parameter optimization of the adjacency matrix’s weighting power (β). A parameter sweep was conducted across β = 1–30, with scale-free topology fit index (R²) and mean connectivity computed for each iteration (Fig. 3A). The optimal β value (β = 30) was selected to simultaneously maximize R² (approaching unity) while preserving biologically meaningful connectivity. Using this threshold, 13,348 genes were partitioned into co-expression modules via dynamic tree-cutting algorithms, yielding two distinct modules (Fig. 3B). The Grey module, representing unassigned genes with non-interpretable expression patterns, was excluded from downstream analyses. Hierarchical clustering of all genes was visualized through a Euclidean distance heatmap (Fig. 3C). Based on the results of the WGCNA module division, cluster plots, box plots, and bar charts were employed to present module gene expression information (Supplementary Figures S1A-S1C). Significant trait-related modules were identified under stringent thresholds (|correlation coefficient| ≥ 0.3; *P* < 0.05) (Fig. 3D), ultimately revealing 200 DEGs.

### Enrichment analysis of biological functions

**Fig. 4:**
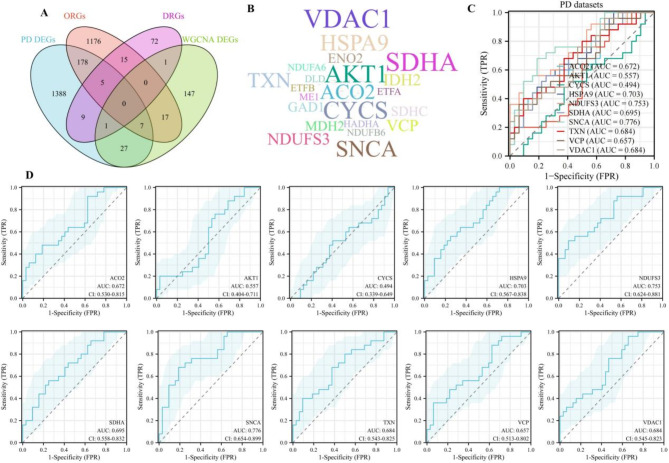
Analysis of the diagnostic value of 10 hub genes. (**A**) Venn diagram of PD DEGs, ORGs, DRGs, and WGCNA DEGs showed that there were 202 overlapping genes between PD DEGs, WGCNA DEGs, and ORGs, 11 overlapping genes between PD DEGs, WGCNA DEGs, and DRGs, 5 overlapping genes between PD DEGs, ORGs, and DRGs. (**B**) Visualize based on the frequency of appearance of hub genes. (**C**) The diagnostic value of 10 hub genes includes GSE20292, GSE20163, and GSE20164 datasets. (**D**) The ROC analysis showed that the respective area under the curves (AUCs) of ACO2, AKT1, CYCS, HSPA9, NDUFS3, SDHA, SNCA, TXN, VCP, and VDAC1 were 0.672 (CI: 0.530–0.815), 0.557 (CI: 0.404–0.711), 0.494 (CI: 0.339–0.649), 0.703 (CI: 0.567–0.838), 0.753 (CI: 0.624–0.881), 0.695 (CI: 0.558–0.832), 0.776 (CI: 0.654–0.899), 0.684 (CI: 0.543–0.825), 0.657 (CI: 0.513–0.802), and 0.684 (CI: 0.545–0.823)

The 218 targets were identified through the intersection of DEGs, ORGs, and DRGs (Fig. 4A). GO analysis was divided into biological processes (BPs), cellular components (CCs), and molecular functions (MFs). The BPs of the DEGs were mainly enriched in response to OS, cellular respiration, cellular response to chemical stress, energy derivation by oxidation of organic compounds, electron transport chain, cellular response to OS, respiratory electron transport chain, ATP metabolic process, oxidative phosphorylation, and mitochondrial ATP synthesis coupled electron transport (Supplementary Figure S2A). The CCs of the DEGs were mainly enriched in mitochondrial matrix, oxidoreductase complex, mitochondrial protein complex, distal axon, respirasome, respiratory chain complex, mitochondrial respirasome, neuron projection terminus, mitochondrial respiratory chain complex I, and NADH dehydrogenase complex (Supplementary Figure S2B). The MFs of the DEGs were mainly enriched in electron transfer activity, oxidoreductase activity, acting on NAD(P)H, NADH dehydrogenase activity, NADH dehydrogenase (ubiquinone) activity, NADH dehydrogenase (quinone) activity, oxidoreductase activity, acting on NAD(P)H, quinone or similar compound as acceptor, lyase activity, antioxidant activity, iron-sulfur cluster binding, and metal cluster binding (Supplementary Figure S2C). The pathways of the DEGs were mainly enriched in Pathways of neurodegeneration- multiple diseases, Parkinson disease, Diabetic cardiomyopathy, Chemical carcinogenesis - reactive oxygen species, Amyotrophic lateral sclerosis, Alzheimer disease, Carbon metabolism, Huntington disease, Citrate cycle (TCA cycle), and Thermogenesis (Supplementary Figure S2D).

### Analysis of PPI networks and identification of hub genes

The STRING database was used to construct the PPI network to identify the interactive relationships between 218 DEGs. A total of 217 nodes, 2035 edges, average node degree of 18.8, avg. local clustering coefficient of 0.445, and PPI enrichment *p*-value < 1 × 10^− 16^ was identified in the PPI network (Supplementary Figure S3A). According to the 12 algorithms, select the following genes as hub genes, namely, ACO2, AKT1, CYCS, HSPA9, NDUFS3, SDHA, SNCA, TXN, VCP, and VDAC1 (Supplementary Figure S3B). Visualize based on the frequency of appearance of hub genes (Fig. 4B).

### Identification and validation of diagnostic feature biomarkers

ROC curves were used to evaluate the diagnostic value of 10 hub genes in PD. Figure 4C and D shows the diagnostic values of the 10 hub genes in the PD datasets. In the validation datasets (GSE49036, GSE24378, GSE49126, and GSE99039), the gene expression level and diagnostic value were further verified (Supplementary Figures S4A-S4D). ROC analysis revealed substantial variability in discrimination ability across genes: ACO2 (AUC = 0.672, 95% CI: 0.530–0.815), AKT1 (0.557, 0.404–0.711), CYCS (0.494, 0.339–0.649), HSPA9 (0.703, 0.567–0.838), NDUFS3 (0.753, 0.624–0.881), SDHA (0.695, 0.558–0.832), SNCA (0.776, 0.654–0.899), TXN (0.684, 0.543–0.825), VCP (0.657, 0.513–0.802), and VDAC1 (0.684, 0.545–0.823).

Following general AUC interpretation guidelines, SNCA demonstrated excellent discrimination (AUC > 0.8), while NDUFS3 showed acceptable discrimination (0.7–0.8). Most genes exhibited poor discrimination (0.5–0.7), with CYCS performing at chance level (AUC ≈ 0.5). Notably, HSPA9 (0.703), SDHA (0.695), TXN (0.684), and VDAC1 (0.684) approached the acceptable range despite their confidence intervals crossing the 0.7 threshold. These variations likely reflect inherent biological variability in gene expression patterns across individuals. While no single gene achieved consistently excellent discrimination, the combination of these hub genes may provide clinical utility for PD identification and progression monitoring through serum protein changes. The relatively wide confidence intervals observed (e.g., SNCA: 0.654–0.899) emphasize the need for larger validation studies to precisely estimate diagnostic accuracy.

### Hub gene and DED-ORGs analysis

Heatmaps and boxplots were used to visualize hub genes and DED-ORGs in the PD datasets (Supplementary Figures S5A, S5B). Supplementary Figure S5C shows the correlations of the hub genes and DED-ORGs in the PD datasets, with most genes showing a positive correlation. The correlation coefficient are shown in Supplementary Table S3. The chromosomal location are shown in Supplementary Figure S5D. The BPs were mainly enriched in aerobic respiration, energy derivation by oxidation of organic compounds, cellular respiration, oxidative phosphorylation, and ATP metabolic process; the CCs were mainly enriched in mitochondrial matrix, respirasome, mitochondrial protein-containing complex, inner mitochondrial membrane protein complex, and respiratory chain complex; the MFs were mainly enriched in iron-sulfur cluster binding, electron transfer activity, 4 iron, 4 sulfur cluster binding, NADH dehydrogenase (ubiquinone) activity, and oxidoreduction-driven active transmembrane transporter activity; the pathway were mainly enriched in Parkinson disease, Pathways of neurodegeneration - multiple diseases, Oxidative phosphorylation, Citrate cycle (TCA cycle), and Apoptosis (Supplementary Figure S5E). The biological functions of 15 genes can be found in Supplementary Table S4.

### Expression of hub genes in MPTP-induced PD mice model

**Fig. 5:**
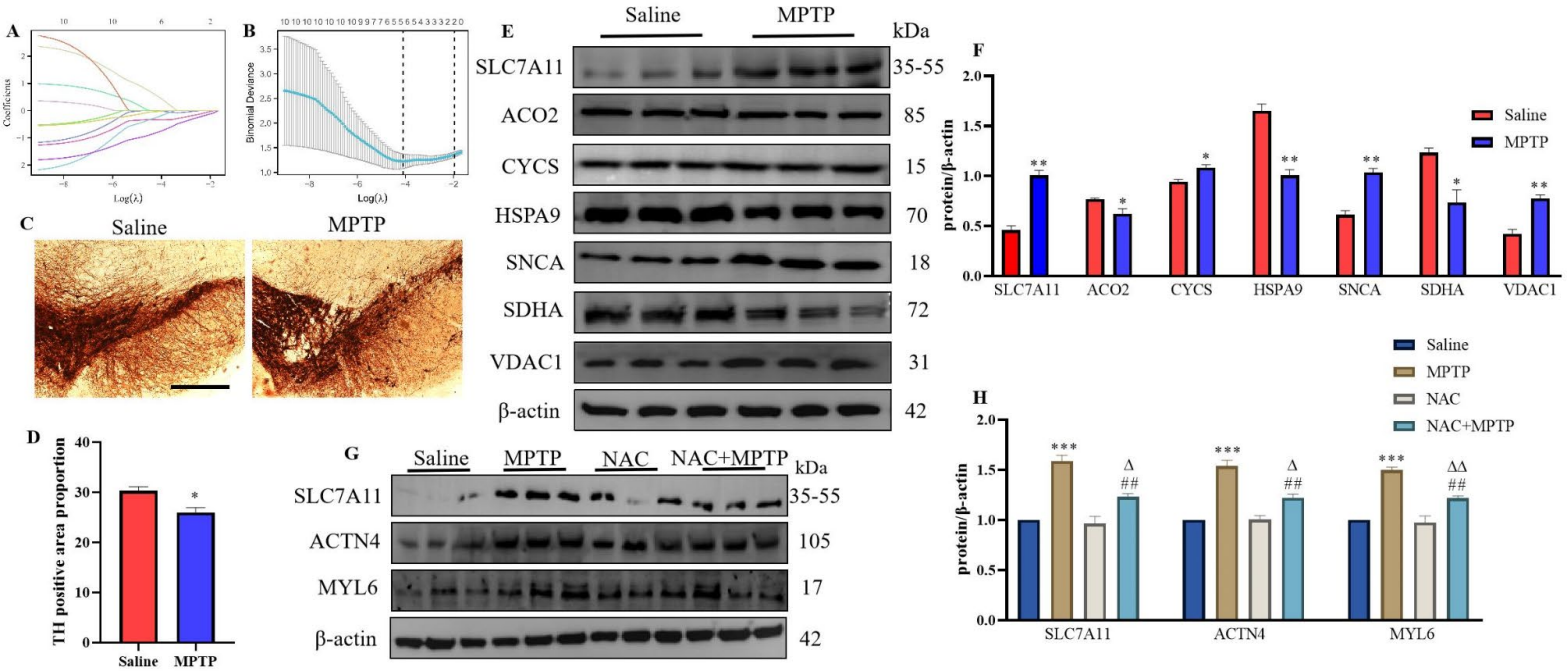
Validation of hub genes expression in the MPTP-induced PD mice model. (**A**), (**B**) Six potential biomarkers were identified using the LASSO regression algorithm. (**C**) TH immunohistochemistry (Scale bar, 100 μm). (**D**) Statistical plot of TH positive area proportion. (**E**) Expression of SLC7A11, ACO2, CYCS, HSPA9, SNCA, SDHA, and VDAC1 proteins in SN in MPTP-induced PD mice model. (**F**) Statistical plot of SLC7A11, ACO2, CYCS, HSPA9, SNCA, SDHA, and VDAC1 proteins expression. (**G**) Expression of disulfidptosis marker proteins SLC7A11, ACTN4, and MYL6 in an MPTP-induced PD mice model treated with the ROS inhibitor NAC. (**H**) Statistical plot of SLC7A11, ACTN4, and MYL6 proteins expression. *n* = 3. Compared with the Saline group, *P < 0.05, **P < 0.01, ***P < 0.001; compared with the MPTP group, ^##^*P* < 0.01; compared with the NAC group, ^∆^*P* < 0.05, ^∆∆^*P* < 0.01

LASSO regression prioritized six candidate biomarkers (ACO2, CYCS, HSPA9, SNCA, SDHA, and VDAC1) with optimal diagnostic performance (Figs. 5 A, 5B). Immunohistochemical analysis revealed significant downregulation of TH expression in the MPTP-treated group compared to Saline group (Fig. 5C and D), confirming successful establishment of the PD mice model. Notably, the disulfidptosis marker protein SLC7A11 showed elevated expression in MPTP-treated mice, indicating disulfidptosis activation in PD pathogenesis. Subsequent analysis of hub gene expression demonstrated differential regulation in MPTP-treated mice, ACO2, HSPA9, and SDHA were significantly downregulated, while CYCS, SNCA, and VDAC1 exhibited upregulation (Fig. 5E and F). Interestingly, GEO dataset analysis revealed reduced RNA expression of these hub genes (Supplementary Figures S5A, S5B). Potential explanations for this discrepancy may include: (1) enhanced translation efficiency, (2) increased mRNA stability with prolonged half-life, and (3) biological negative feedback regulation mechanisms that could decouple transcriptional and translational outputs.

Notably, pharmacological inhibition of ROS using NAC significantly mitigated MPTP-induced disulfidptosis signatures. While MPTP elevated SLC7A11, ACTN4, and MYL6 protein expression, NAC co-treatment effectively normalized these markers (Fig. 5G and H). Collectively, these data establish ROS as a critical mediator of disulfidptosis in PD models, highlighting oxidative stress modulation as a viable therapeutic strategy.

### Expression of DED-ORGs in MPTP-induced PD mice model

**Fig. 6:**
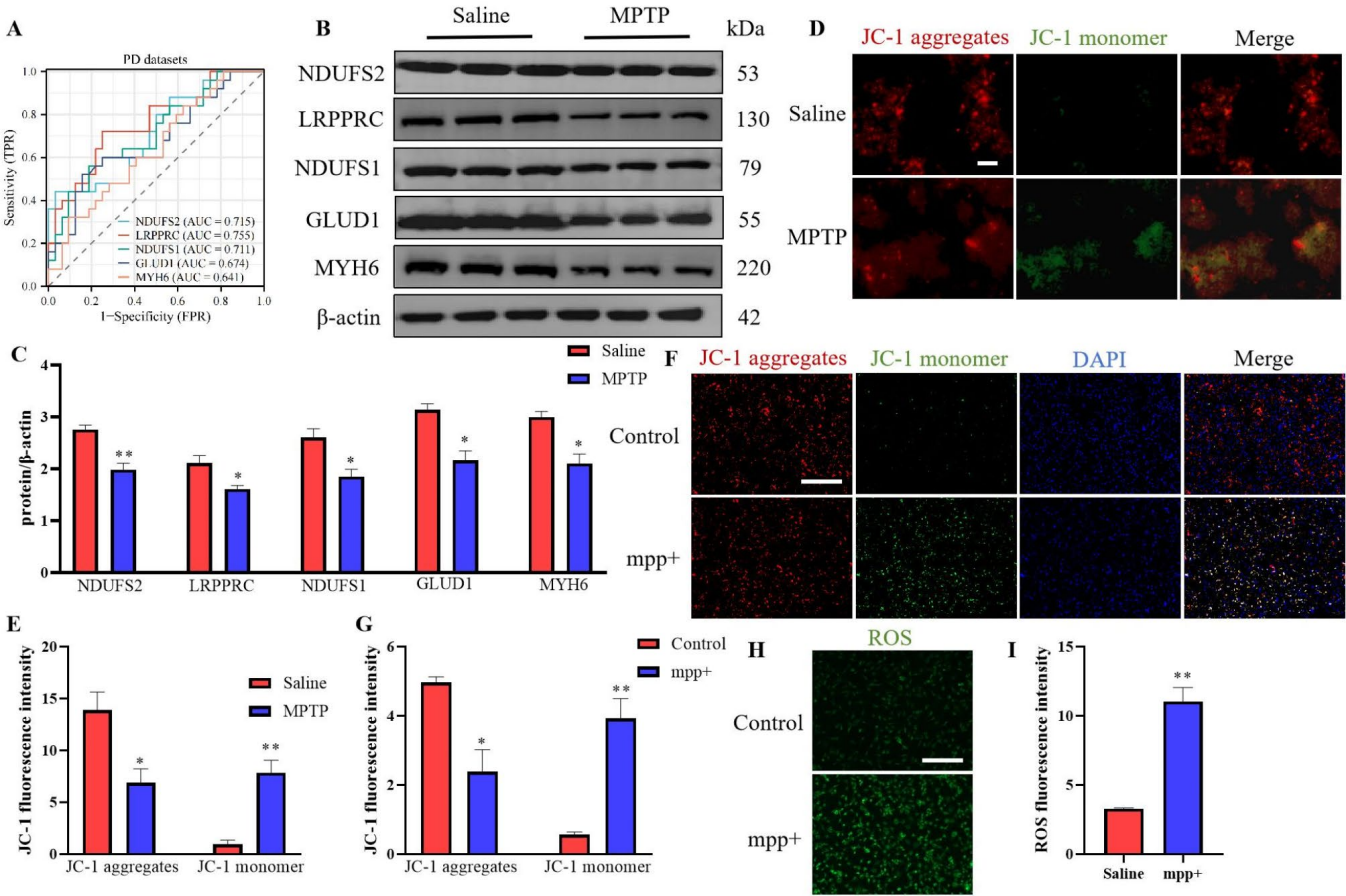
Validation of DED-ORGs expression and mitochondrial dysfunction in mptp-induced pd mice and SH-SY5Y cell models. (**A**) ROC analysis of mitochondrial biomarkers (NDUFS2, LRPPRC, NDUFS1, GLUD1, MYH6) for PD diagnosis. (**B**) Representative Western blot images of DED-ORGs protein expression in SN tissue from MPTP-treated mice. (**C**) Quantitative analysis of DED-ORGs protein levels in SN. (**D**) JC-1 staining (red: aggregates; green: monomers) in SN mitochondria (Scale bar, 20 μm). (**E**) Quantification of JC-1 fluorescence intensity ratio (aggregates/monomers) in SN. (**F**) JC-1 staining in SH-SY5Y cells treated with 1 mmol/L MPP+ (Scale bar, 50 μm). (**G**) Quantification of JC-1 fluorescence intensity ratio in SH-SY5Y cells. (**H**) ROS detection (green fluorescence) in MPP+-treated SH-SY5Y cells (Scale bar, 50 μm). (**I**) Quantitative analysis of ROS fluorescence intensity in SH-SY5Y cells. *n* = 3. Compared with the Saline group, *P < 0.05, **P < 0.01

ROC curve analysis revealed substantial variability in the diagnostic performance of mitochondrial biomarkers, with LRPPRC demonstrating the highest discrimination ability (AUC = 0.755, 95% CI: 0.627–0.883), followed by NDUFS2 (0.715, 0.579–0.851) and NDUFS1 (0.711, 0.575–0.848), while GLUD1 (0.674, 0.530–0.819) and MYH6 (0.641, 0.497–0.786) showed more modest performance (Fig. 6A, Supplementary Figures S4E). Following AUC interpretation guidelines, LRPPRC reached acceptable discrimination (0.7–0.8 range), with its confidence interval approaching the excellent category (> 0.8). Notably, NDUFS2 and NDUFS1 approached the acceptable threshold (0.7) but their CIs spanned both poor and acceptable ranges. This pattern suggests biological heterogeneity in mitochondrial gene expression profiles, where certain biomarkers like LRPPRC may have particular utility in clinical stratification despite the overall variability observed across targets.

Subsequent validation demonstrated significant downregulation of these hub genes in MPTP-treated mice compared to Saline group (Fig. 6B and C), confirming their association with PD pathology. Consistent with established mechanisms linking mitochondrial impairment to redox imbalance [24], our findings suggest a pathogenic cascade: disrupted mitochondrial dynamics and mitophagy → ROS overproduction → oxidative stress → disulfidptosis. This pathway was experimentally validated through mitochondrial membrane potential (ΔΨm) assessments. In SN tissue, saline controls exhibited preserved ΔΨm evidenced by JC-1 aggregate red fluorescence, while MPTP treatment induced significant depolarization (increased green monomeric fluorescence; Fig. 6D and E). Parallel results emerged in SH-SY5Y cell models: untreated cells maintained normal ΔΨm (red fluorescence), whereas 1 mmol/L MPP + exposure caused ΔΨm collapse (green fluorescence intensification; Fig. 6F and G). Quantitative analysis confirmed increased JC-1-stained cell populations post-MPP + exposure (Fig. 6H and I), indicative of progressive mitochondrial dysfunction.

These results establish a mechanistic connection between MPTP/MPP+-induced mitochondrial impairment and cellular pathogenesis. The observed ΔΨm dissipation directly correlates with OS amplification and disulfidptosis activation, proposing a unified pathway where mitochondrial failure initiates redox dysregulation and subsequent non-apoptotic cell death in PD models.

### Single cell type analysis

We also analyzed the expression of genes (AUC>0.7) in different cells of the brain during fetal development and adulthood, including HSPA9, SNCA, NDUFS1, NDUFS2, NDUFS3, and LRPPRC. For example, during fetal development, HSPA9, NDUFS1, NDUFS2, and LRPPRC are highly expressed in the pericytes, SNCA is highly expressed in the erythroid cells, and NDUFS3 is highly expressed in the proliferating glia; during adulthood, HSPA9 is highly expressed in the astrocytes, SNCA, NDUFS1, and LRPPRC are highly expressed in the neurons, NDUFS2 is highly expressed in the oligodendrocytes, and NDUFS3 is highly expressed in the granule cells (Supplementary Figure S6).

### Molecular Docking

**Fig. 7:**
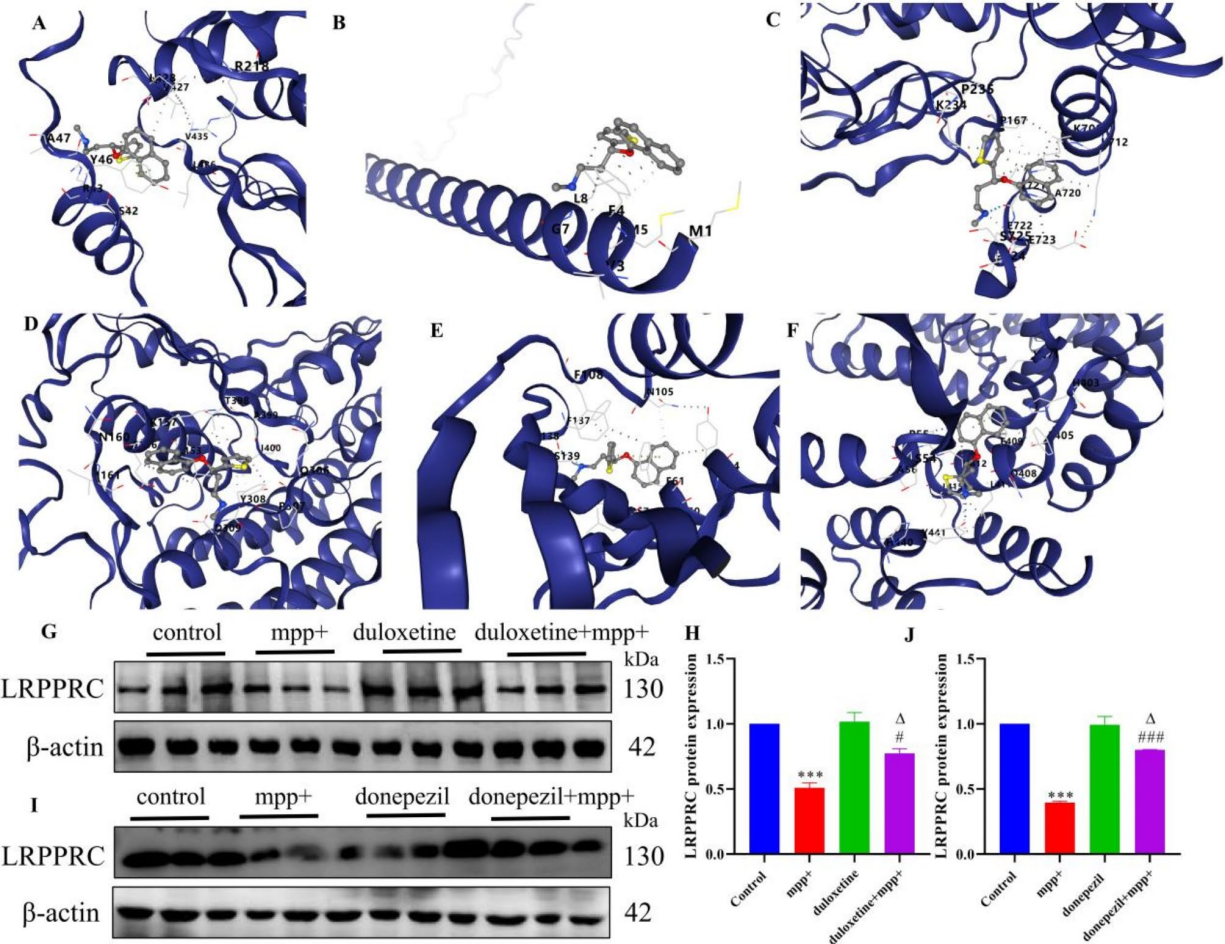
Molecular docking analysis docking sites, including (**A**) HSPA9 protein and Duloxetine docking, (**B**) SNCA protein and Duloxetine docking, (**C**) NDUFS1 protein and Duloxetine docking, (**D**) NDUFS2 protein and Duloxetine docking, (**E**) NDUFS3 protein and Duloxetine docking, and (**F**) LRPPRC protein and Duloxetine docking. (**G**) The expression of LRPPRC protein in a Duloxetine-treated MPP+-induced PD cell model. (**H**) Statistical plot of LRPPRC protein expression. (**I**) The expression of LRPPRC protein in a Donepezil-treated MPP+-induced PD cell model. (**J**) Statistical plot of LRPPRC protein expression. *n* = 3. Compared with the Control group, ***P < 0.001; compared with the MPP + group, ^#^*P* < 0.05, ^###^*P* < 0.001; compared with the Duloxetine or Donepezil group, ^∆^*P* < 0.05

Our multi-phase drug discovery pipeline began with systematic screening of PD-modifying agents in the Malacards database (Supplementary Table S5), prioritizing compounds with established clinical efficacy. Focused analysis on Phase IV therapeutics (Supplementary Table S6) revealed Duloxetine and Donepezil as promising candidates for mechanistic investigation. Using Autodock Vina v1.2.2, we characterized the binding dynamics between these drugs and six PD-related targets. Computational modeling demonstrated stable ligand-receptor interactions, with detailed binding poses visualized in Supplementary Figure S7 and quantified through binding energy calculations (Fig. 7A and F, Supplementary Figure S8). LRPPRC exhibited favorable binding energies of -7.6 kcal/mol (Duloxetine) and − 8.7 kcal/mol (Donepezil) (Lower binding energy values indicate stronger molecular interactions and enhanced complex stability). Specific interacting residues critical for drug-target stabilization are cataloged in Table 2.

In MPP+-induced PD cell models, pathological upregulation of LRPPRC expression confirmed disease-associated mitochondrial dysregulation. Therapeutic intervention with Duloxetine or Donepezil significantly normalized LRPPRC levels (Fig. 7G and J). These results validate both the computational predictions and therapeutic potential of these repurposed agents in mitigating PD-associated molecular pathology.

**Table 2:** Vina score and amino acid sites of drug molecular Docking with protein

Protein	Drug	Vina score ( kcal/mol)	Amino acid sites
HSPA9	Duloxetine	−7.1	Chain A: ASN31 GLY32 LEU33 SER34 HIS35 PHE38 ARG39 SER42 ARG43 TYR46 ALA47 SER48 ILE51 ARG218 GLU263 GLN424 VAL427 LEU428 ALA429 GLY430 VAL432 THR433 ASP434 VAL435 LEU436 LEU437 LEU438 ILE464 PRO465 ALA526
SNCA		−4.4	Chain A: MET1 ASP2 VAL3 PHE4 MET5 LYS6 GLY7 LEU8 SER9 ALA11 LYS12
NDUFS1		−7.6	Chain A: PRO167 LEU168 LYS170 SER233 LYS234 PRO235 TYR236 ALA237 PHE238 THR239 VAL270 MET271 TYR294 ASP295 LYS298 ARG299 GLN705 THR706 LYS709 LYS712 ALA713 ALA720 VAL721 GLU722 GLU723 PRO724 SER725
NDUFS2		−6.0	Chain A: LEU153 ALA154 GLU156 LYS157 ASN160 ILE161 ARG162 PRO163 GLN168 THR305 GLN306 PRO307 TYR308 ASP309 ASP312 TYR397 THR398 ALA399 ILE400 TYR410 LYS423 ILE424 LYS425 ALA426
NDUFS3		−6.9	Chain A: ASN51 ALA54 LYS56 GLN57 LEU58 ALA60 PHE61 TYR64 PHE81 ASN82 ASN105 ALA106 GLN107 PHE108 SER134 PHE137 ASN138 SER139 ARG140
LRPPRC		−7.6	Chain A: LEU52 LEU53 SER54 PRO55 ALA56 PRO369 SER370 VAL371 SER374 HIS403 SER404 PHE405 GLN408 PHE409 LEU411 HIS412 LEU415 PHE435 PRO436 ARG438 HIS440 TYR441
HSPA9	Donepezil	−8.4	Chain A: HIS35 PHE38 ARG39 VAL41 SER42 ARG43 ASP45 TYR46 ALA47 SER48 GLU49 ALA50 ILE51 ARG218 LYS259 GLY260 VAL261 GLN424 VAL427 LEU428 ALA429 GLY430 VAL432 THR433 ASP434 VAL435 LEU436 LEU437 LEU438 ASP439 THR463 ILE464 PRO465 THR466 ASP525 ALA526
SNCA		−5.5	Chain A: ILE88 ALA89 ALA90 THR92 GLY93 PHE94 VAL95 LYS96 LYS97 ASP98 GLN99 LEU100 GLY101 LYS102 ASN103 GLU104 GLU105
NDUFS1		−8.6	Chain A: ASP162 LYS163 ASN164 ILE165 GLY166 PRO167 VAL169 LYS170 THR171 LYS234 PRO235 ALA237 PHE238 THR239 GLU269 TYR294 ASP295 LYS298 ARG299 GLN705 THR706 LYS709 LYS712 ALA713 ALA720 VAL721 GLU722 GLU723 PRO724 SER725
NDUFS2		−7.4	Chain A: LEU153 GLU156 LYS157 ASN160 ILE161 ARG162 PRO163 GLN168 LEU284 GLY287 PHE288 LEU293 ILE298 TRP300 LEU302 THR305 GLN306 PRO307 TYR308 ASP309 ASP312 TYR397 THR398 ALA399 GLU406 TYR410 LYS423 ILE424 LYS425
NDUFS3		−7.6	Chain A: ARG48 ASN51 ALA54 GLN57 LEU58 ALA60 PHE61 TYR64 GLU67 PHE81 ASN82 GLU83 ASN105 ALA106 PHE108 SER134 PHE137 ASN138 SER139 ARG140
LRPPRC		−8.7	Chain A: GLY51 LEU52 LEU53 SER54 PRO55 ALA56 LEU58 TYR59 PRO369 VAL371 SER374 HIS403 SER404 PHE405 GLN408 PHE409 LEU411 HIS412 LEU415 PHE435 PRO436 ARG438 HIS440 TYR441

## Discussion

PD is a chronic and progressive neurodegeneration condition characterized by the loss of dopamine neurons in the SN. The reason for the death of these neurons remains unclear; however, studies have demonstrated the potential involvement of mitochondria, the endoplasmic reticulum, α-synuclein, and dopamine levels in contributing to cellular OS as well as PD symptoms [25]. In the MPTP mice model of PD, time-course experiments indicate that OS is an early event that may directly cause the death of some dopaminergic neurons. In this model, it appears that OS may also play a more significant role in the indirect loss of dopaminergic neurons by activating intracellular molecular pathways related to cell death. As the neurodegenerative process progresses in the MPTP mice model, indicators of neuroinflammation emerge, such as the activation of microglia. This activation enhances the level of OS affecting neighboring compromised neurons, thereby contributing to their demise [26].

This study uncovers the crucial role of OS-induced disulfidptosis in the pathology of PD. Through the use of an MPTP-induced PD mouse model, we observed a significant upregulation in the expression of the disulfidptosis marker protein SLC7A11, which could be effectively reversed by the ROS inhibitor NAC. This finding not only confirms the activation of disulfidptosis in PD but also suggests that it may lead to cytoskeleton collapse through abnormal disulfide bond accumulation, ultimately triggering neuronal death. Notably, NAC intervention significantly reduced the expression of ACTN4 and MYL6, indicating that targeting redox imbalance could emerge as a novel therapeutic strategy to inhibit disulfidptosis. Unlike previous studies [27–29], our data link disulfidptosis to mitochondrial dysfunction and energy metabolism disorders in PD, providing a new perspective on the molecular mechanisms of PD. As the primary source of ROS, mitochondrial dysfunction has been extensively reported in PD [30–32]. Our research found that MPTP treatment resulted in a significant decrease in mitochondrial membrane potential (ΔΨm), which was closely associated with the downregulation of mitochondrial complex I subunits such as NDUFS1 and NDUFS2. Abnormalities in these genes may exacerbate ROS production by disrupting the electron transport chain, creating a vicious cycle of “oxidative stress-mitochondrial damage.” Furthermore, single-cell analysis revealed the specific distribution of HSPA9 and LRPPRC in neurons and glial cells, suggesting their differential regulatory roles in various brain cell types. Notably, the strong binding affinity of LRPPRC to clinical drugs (such as donepezil) and its expression regulation in the MPP + model provide experimental evidence for the development of therapeutic regimens targeting the mitochondrial-oxidative stress axis. These discoveries not only deepen our understanding of the pathological mechanisms of PD but also open up a new direction for precision treatment based on disulfidptosis.

In order to further explore the biomarkers of OS-induced disulfidptosis, hub genes were screened using cytohubba, with an AUC > 0.7 selected as the biomarker for PD, namely, HSPA9, SNCA, NDUFS1, NDUFS2, NDUFS3, and LRPPRC. These genes were mainly enriched in pathways related to aerobic respiration, oxidative phosphorylation, and ATP metabolic process. HSPA9 expression has been shown to be regulated by different cellular stresses such as glucose deprivation, oxidative injury, ionizing radiation, and calorie restriction [33]. Low levels of HSPA9 were first observed in post-mortem brains of PD patients and individuals suffering from Parkinsonism due to manganese exposure [34]. Manganese and rotenone, recognized environmental factors in PD, directly target mitochondria and result in a reduction of HSPA9 levels in the mitochondrial matrix [35]. Consequently, HSPA9 depletion in the brain could exacerbate PD symptoms following exposure to environmental toxicants. Additionally, studies have demonstrated that HSPA9 interacts with proteins associated with familial forms of PD. Direct interactions between HSPA9 and both α-synuclein and DJ-1 have been described, albeit without functional elucidation [36]. Consistent with this, aggregated α-synuclein can bind to HSPA9 [36], potentially impeding its normal function and leading to proteasomal degradation. This may help explain the toxicity of α-synuclein towards mitochondria [37]. In this study, it was found that the MPTP-induced PD mice model exhibited decreased levels of HSPA9, which may be a contributing factor in the pathophysiology of PD.

As the primary component of LBs, α-synuclein is a well-known contributor to the pathogenesis of PD, with duplications, triplications, and point mutations in its N-terminal region (A30P, A53T, and E46K) linked to familial PD [38, 39]. The accumulation of α-synuclein can exacerbate OS through its direct interaction with mitochondrial membrane proteins [40]. In our study, the MPTP-induced PD mice model exhibited high expression of SNCA protein, which is implicated in OS and disulfidptosis processes. The over-expression or misfolding of α-synuclein increases the production of ROS [41] and increases the cell’s sensitivity to OS [42, 43]. SNCA transgenic mice show increased susceptibility to MPTP and 6-OHDA [44, 45]. Dopaminergic neurons derived from induced pluripotent stem cells (iPSCs) of PD patients with SNCA triplication exhibit high levels of OS markers and enhanced susceptibility to H_2_O_2_, indicating that excess α-synuclein fundamentally alters the balance between ROS production and antioxidant activity in dopaminergic neurons [46]. Furthermore, the accumulation of α-synuclein within the inner mitochondrial membrane inhibits the activity of complex I, leading to mitochondrial dysfunction and increased OS [47]. As an agonist of toll-like receptor 2, oligomeric α-synuclein has the potential to activate microglia [48], resulting in elevated ROS production, followed by the secretion of tumor necrosis factor-α (TNF-α), interleukin-1β (IL-1β), and IL-6 [49, 50]. Notably, the increased ROS levels may contribute to the aggregation of α-synuclein [50], which in turn exacerbates OS, creating another neurotoxic vicious cycle.

NDUFS1, the largest core subunit encoded by nuclear genes, is a component of the complex containing eight iron-sulfur chains and is responsible for the oxidation of NADH [51, 52]. Proton translocation across the inner mitochondrial membrane generates an electrochemical gradient that drives ATP production in complex V. As a byproduct of simultaneous ATP production, ROS are also generated in several mitochondrial complexes, mainly complex I. A previous study demonstrated that Ndufs1 knockdown in neurons resulted in impaired oxygen consumption and increased mitochondrial ROS, and upregulated NDUFS1 expression in astrocytes also decreased ROS generation [53]. In skin fibroblasts obtained from patients with the NDUFS1 mutation, a high level of OS was found, accompanied by a decrease in complex I activity, impaired oxygen consumption, and increased glycolysis [54]. As the starting point of the mitochondrial ETC, NDUFS1 is a dual “engine” for ATP and ROS production and plays a vital role in metabolic reprogramming, OS, and cell apoptosis in several diseases [55]. NDUFS2-knockout mice displayed progressive, axon-first, levodopa-responsive parkinsonism, resembling that seen in humans [56]. NDUFS3-deficient cells revealed that it is possible to systematically induce mitochondrial dysfunction by gene silencing NDUFS3 expression, which is known to be one of the precursor subunits in the mitochondrial complex I assembly [57]. NDUFS1, NDUFS2, and NDUFS3 are closely related to mitochondrial energy generation, and their abnormalities may lead to insufficient energy within mitochondria, which can cause functional damage to nerve cells and accelerate their death, ultimately leading to symptoms of PD. Research on NDUFS1, NDUFS2, and NDUFS3 can help reveal the molecular mechanisms of PD and may provide new targets for treating the disease. For example, improving mitochondrial function or enhancing antioxidant capacity may be potential intervention strategies.

LRPPRC functions as a crucial posttranscriptional regulator of mitochondrial DNA expression and is essential for the polyadenylation and coordination of mt-mRNA translation [58]. Research indicates that LRPPRC and NDUFS1 are resistant to disulfidptosis, and their inactivation works synergistically with glucose deprivation to induce cell death [59]. Within mitochondria, LRPPRC interacts with the translation initiation factor 4E (eIF4E) to regulate the export, stability, and translocation of mRNA [60]. In conjunction with Parkin, LRPPRC potentially contributes to the pathogenesis of PD [61]. LRPPRC levels are significantly reduced in PINK1-deficient dopaminergic neuronal cells, and over-expression of LRPPRC enhances complex IV activity, suggesting that PINK1 modulates complex IV activity through interactions with LRPPRC [62]. Specific minigene assays targeting PD-related genetic variants demonstrate that intronic variants of LRPPRC affect pre-messenger RNA splicing by regulating the inclusion of corresponding exons, and this phenomenon is linked to the risk of PD and associated disorders [63, 64]. These findings illustrate that LRPPRC may play a significant role in the pathogenesis of PD and present a potential therapeutic target for the disease. The development of appropriate animal models will be of importance in understanding the role of LRPPRC in PD progression.

Although this study provides novel insights into the role of disulfidptosis in the pathogenesis of PD, several limitations still deserve consideration. In terms of sample size and data source, the sample size in each group of the experimental validation cohort (such as the MPTP-induced PD mouse model) is relatively small (*n* = 3), which may limit the statistical power and generalizability of the research findings. The publicly available datasets (such as GEO) used for biomarker discovery and validation have incomplete clinical metadata (such as disease stage and medication history) and heterogeneity in tissue sources (such as post-mortem brain and peripheral blood), which may introduce biases and necessitate cautious interpretation of the results. Regarding the biological complexity of disulfidptosis, although key mitochondrial and oxidative stress-related genes (such as NDUFS1 and LRPPRC) have been identified as the core of disulfidptosis, the regulatory network may extend beyond actin cytoskeletal proteins. Other pathways, such as redox-sensitive kinases or metabolic enzymes, may also be involved in disulfide stress, but they were not explored in this study. As for the translational challenges of blood-based biomarkers, although peripheral biomarkers (such as SNCA and LRPPRC) have shown diagnostic potential in blood samples (GSE49126 and GSE99039), their clinical utility has not been validated yet. The discordance of blood-brain transcription and confounding factors (such as systemic inflammation) require large-scale longitudinal studies to confirm their specificity and sensitivity for the early detection of PD. In terms of mechanism, the interaction between disulfidptosis and other cell death pathways (such as ferroptosis and apoptosis) in PD remains unclear. Future work should clarify the crosstalk mechanism and identify therapeutic targets with minimal off-pathway effects.

To address these limitations, we propose to expand preclinical models by incorporating human induced pluripotent stem cell (iPSC)-derived neurons and non-human primates to enhance translational relevance; conduct multi-omics integration (proteomics and metabolomics) to discover novel regulators of disulfidptosis; and carry out prospective clinical trials to validate blood-based biomarkers and evaluate the regulatory effect of NAC or repurposed drugs (such as Duloxetine) on disulfidptosis in PD patients. These improvements will deepen our understanding of disulfidptosis and accelerate its translation into diagnostic and therapeutic strategies for PD.

## Electronic supplementary material

Below is the link to the electronic supplementary material.

Supplementary Material 1

Supplementary Material 2

Supplementary Material 3

## Data Availability

All data and material generated or analyzed during this study are included in this published article [and its supplementary information files].
