# Supplementary material for: Target oxidative stress-induced disulfidptosis: novel therapeutic avenues in Parkinson’s disease
Source: Mol Brain. 2025 Apr 4;18:29. doi: 10.1186/s13041-025-01200-2 (PMC11971801; doi:10.1186/s13041-025-01200-2)
Supplement: Supplementary file 2 — Supplementary Material 2 [file 13041_2025_1200_MOESM2_ESM.docx]

| SLC7A11 | 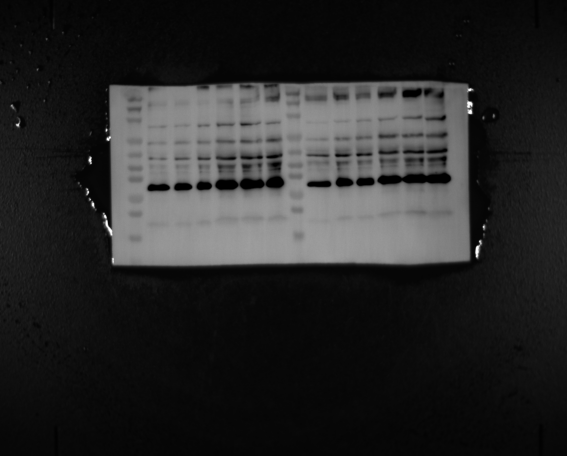 | ACO2 | 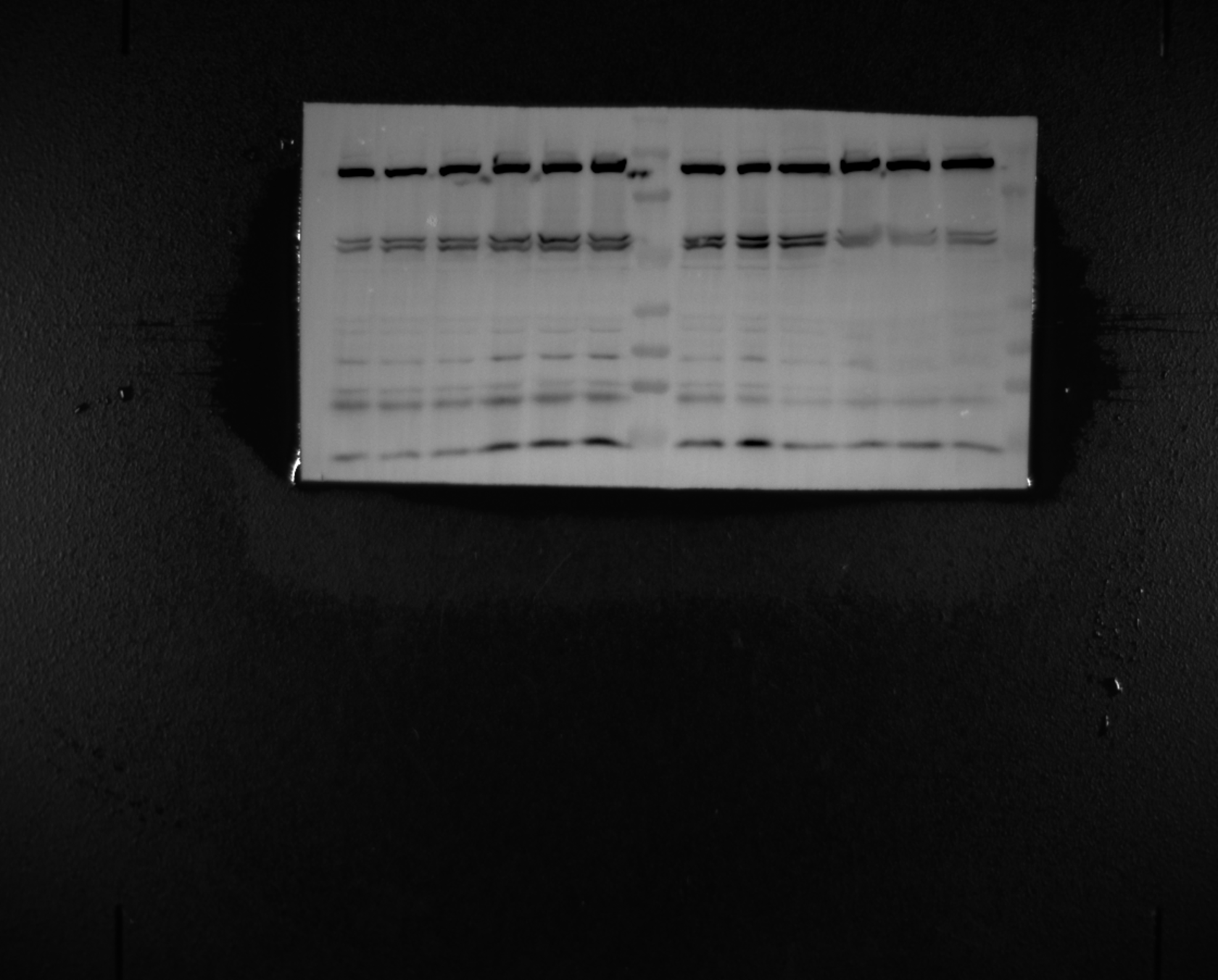 |
| --- | --- | --- | --- |
| CYCS | 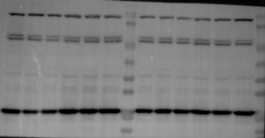 | HSPA9 | 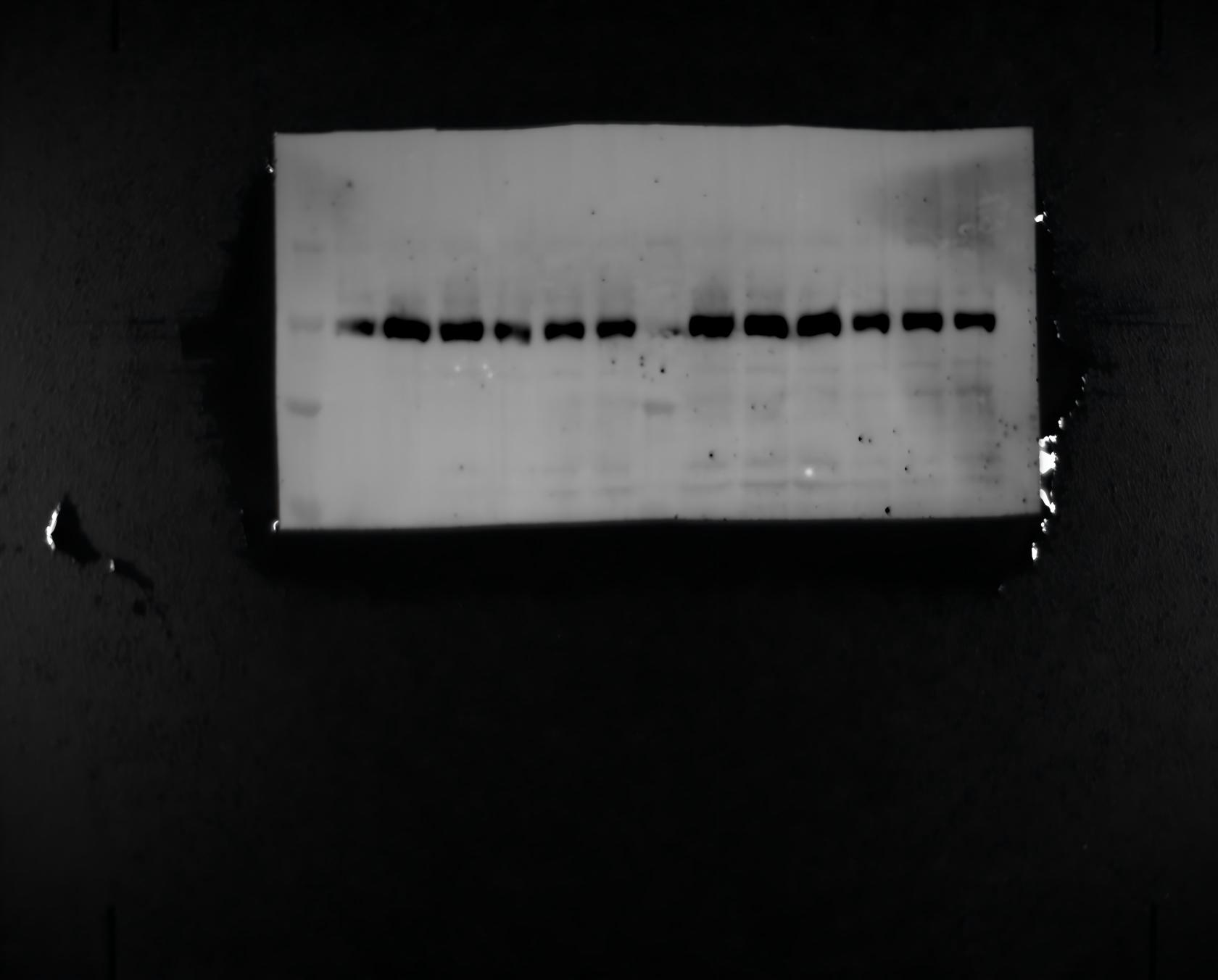 |
| SNCA | 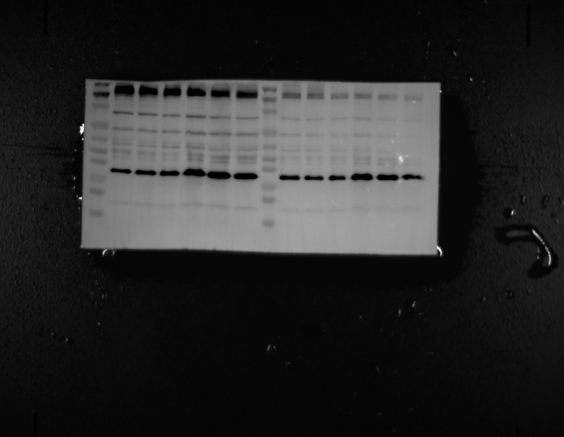 | SDHA | 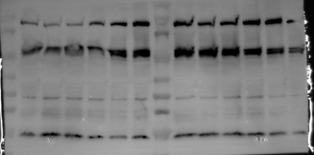 |
| VDAC1 | 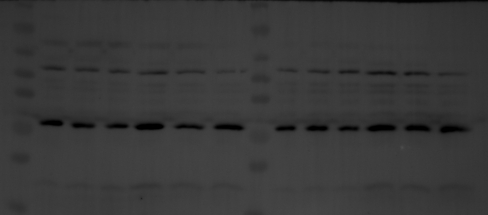 | β-actin | 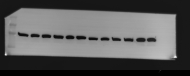 |
| NDUFS2 | 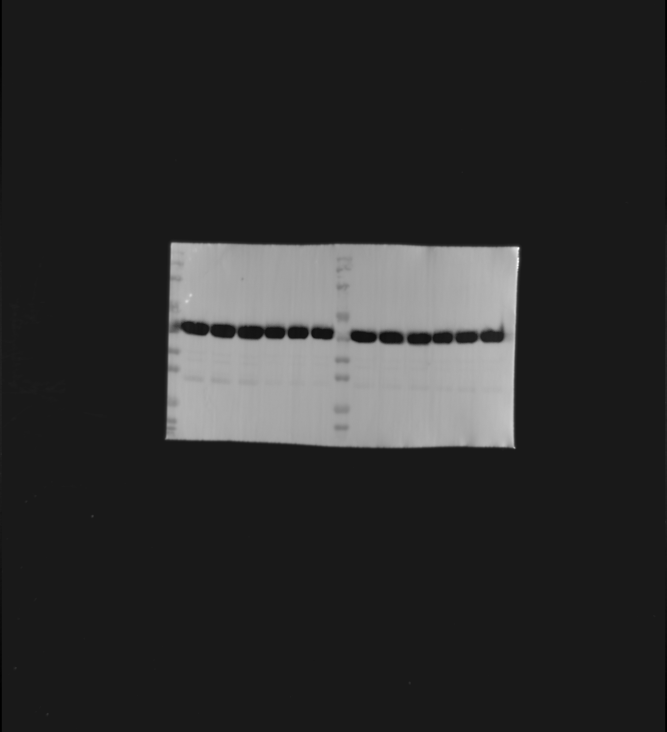 | LRPPRC | 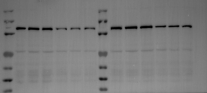 |
| NDUFS1 | 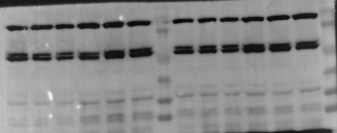 | GLUD1 | 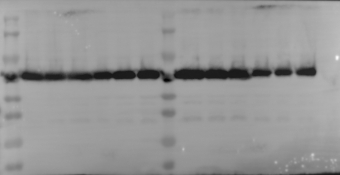 |
| MYH6 | 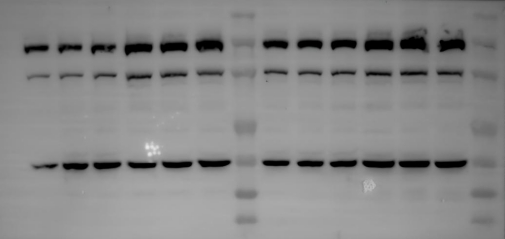 | β-actin | 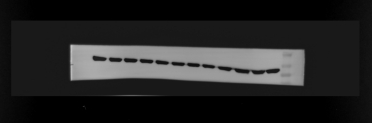 |
| SLC7A11 | 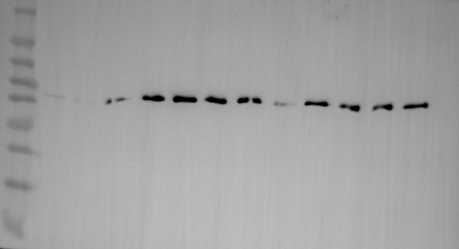 | ACTN4 | 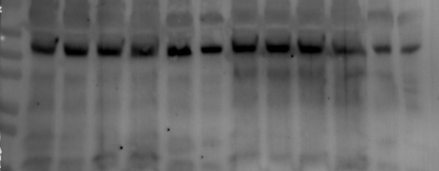 |
| MYL6 | 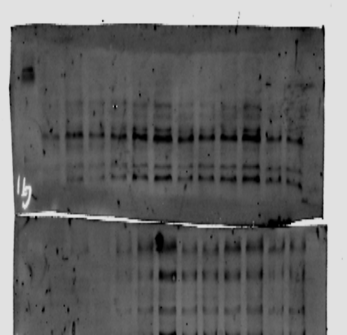 | β-actin | 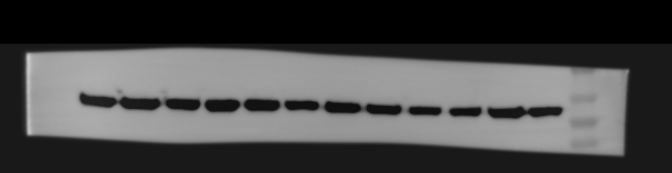 |
| LRPPRC | 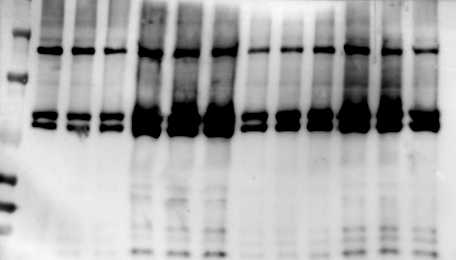 | β-actin | 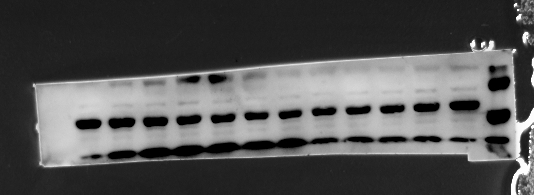 |
| LRPPRC | 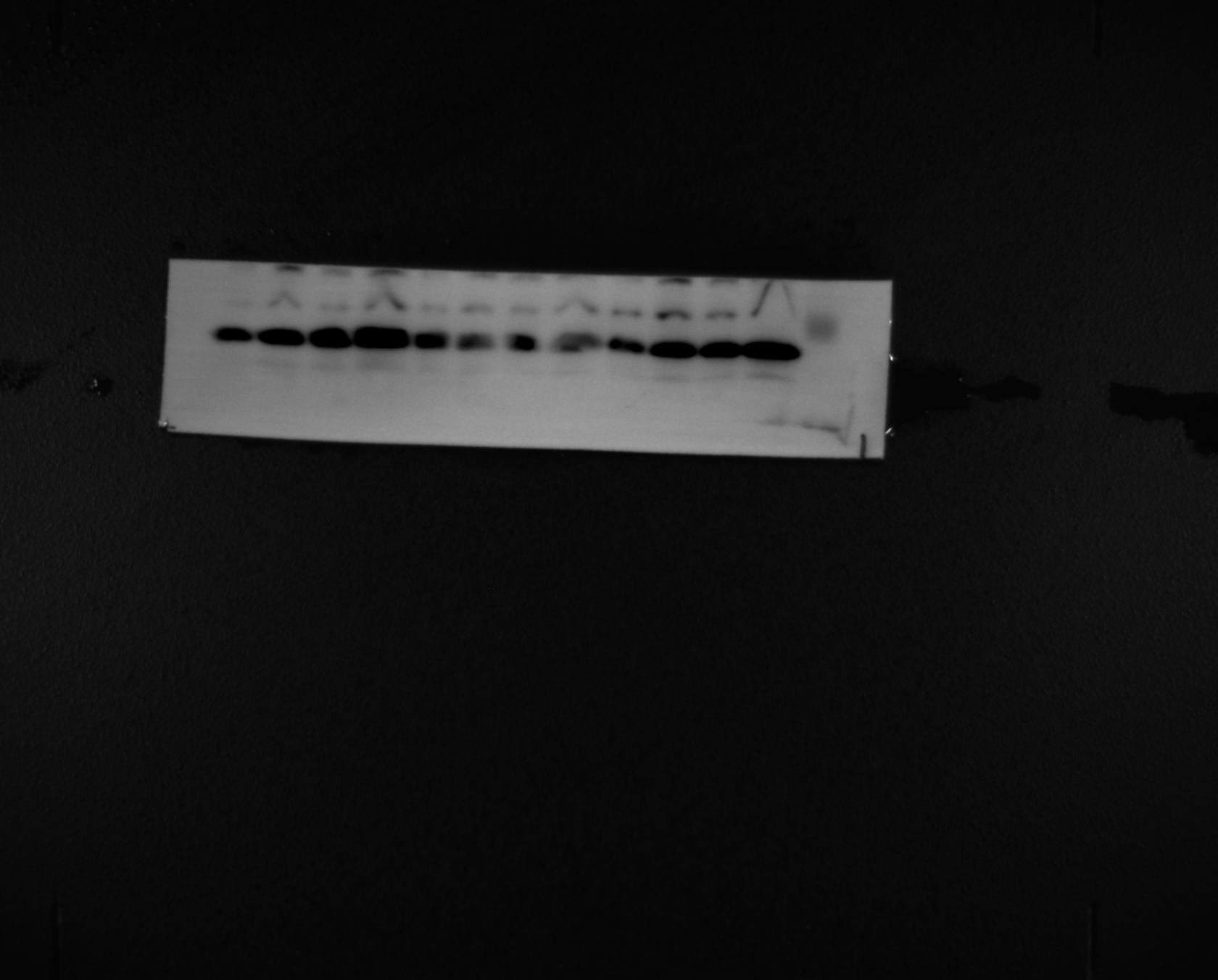 | β-actin | 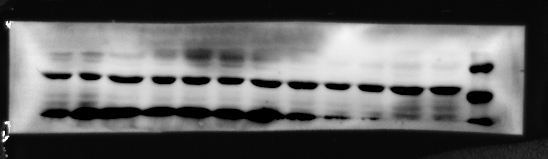 |
