# Supplementary material for: Target oxidative stress-induced disulfidptosis: novel therapeutic avenues in Parkinson’s disease
Source: Mol Brain. 2025 Apr 4;18:29. doi: 10.1186/s13041-025-01200-2 (PMC11971801; doi:10.1186/s13041-025-01200-2)
Supplement: Supplementary file 3 — Supplementary Material 3 [file 13041_2025_1200_MOESM3_ESM.docx]

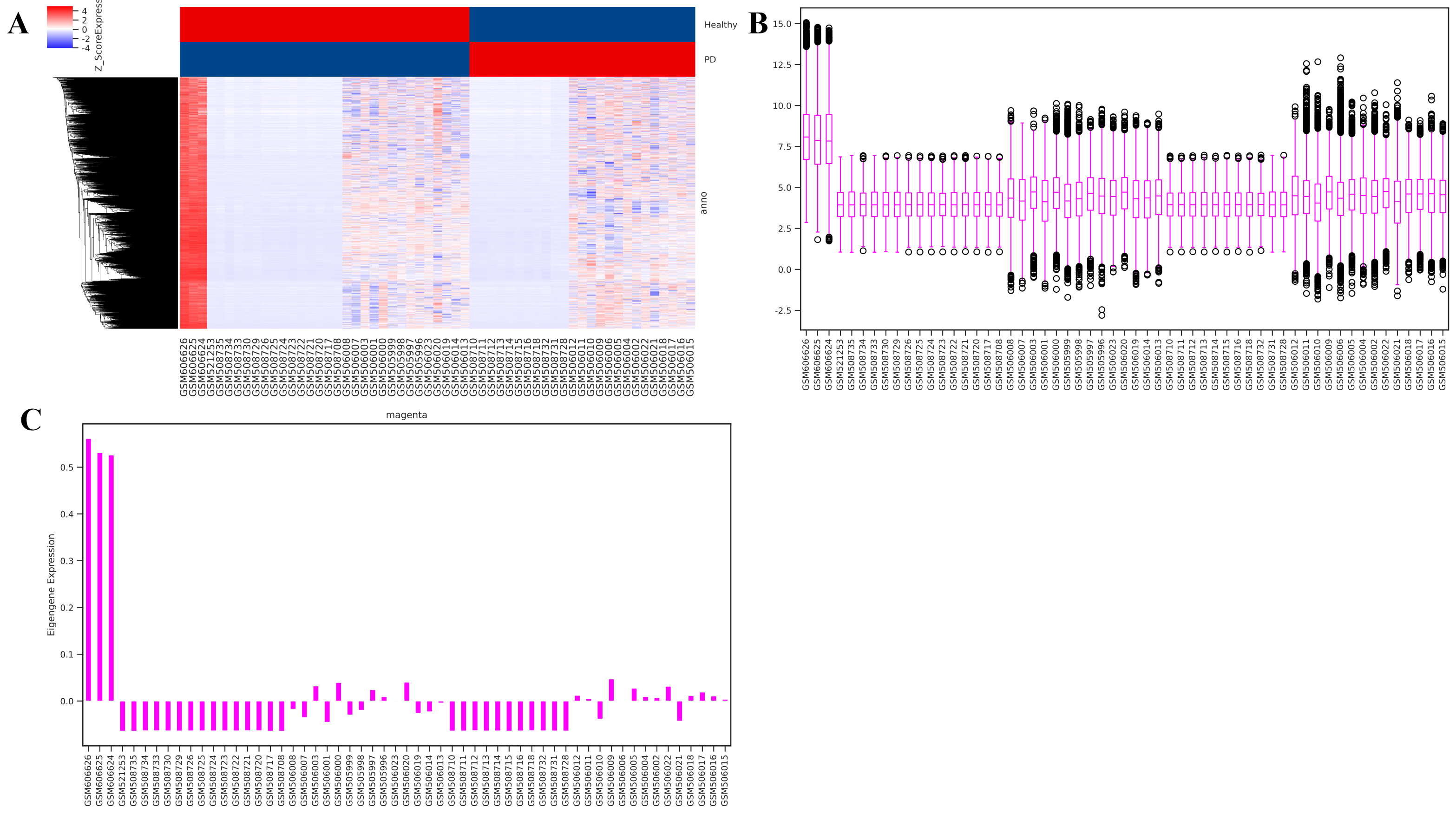


Figure S1 Clustering module analysis. Based on the division results of WGCNA modules, (A) heatmap, (B) box plot, and (C) column plot are used to display module gene expression information in the PD datasets.


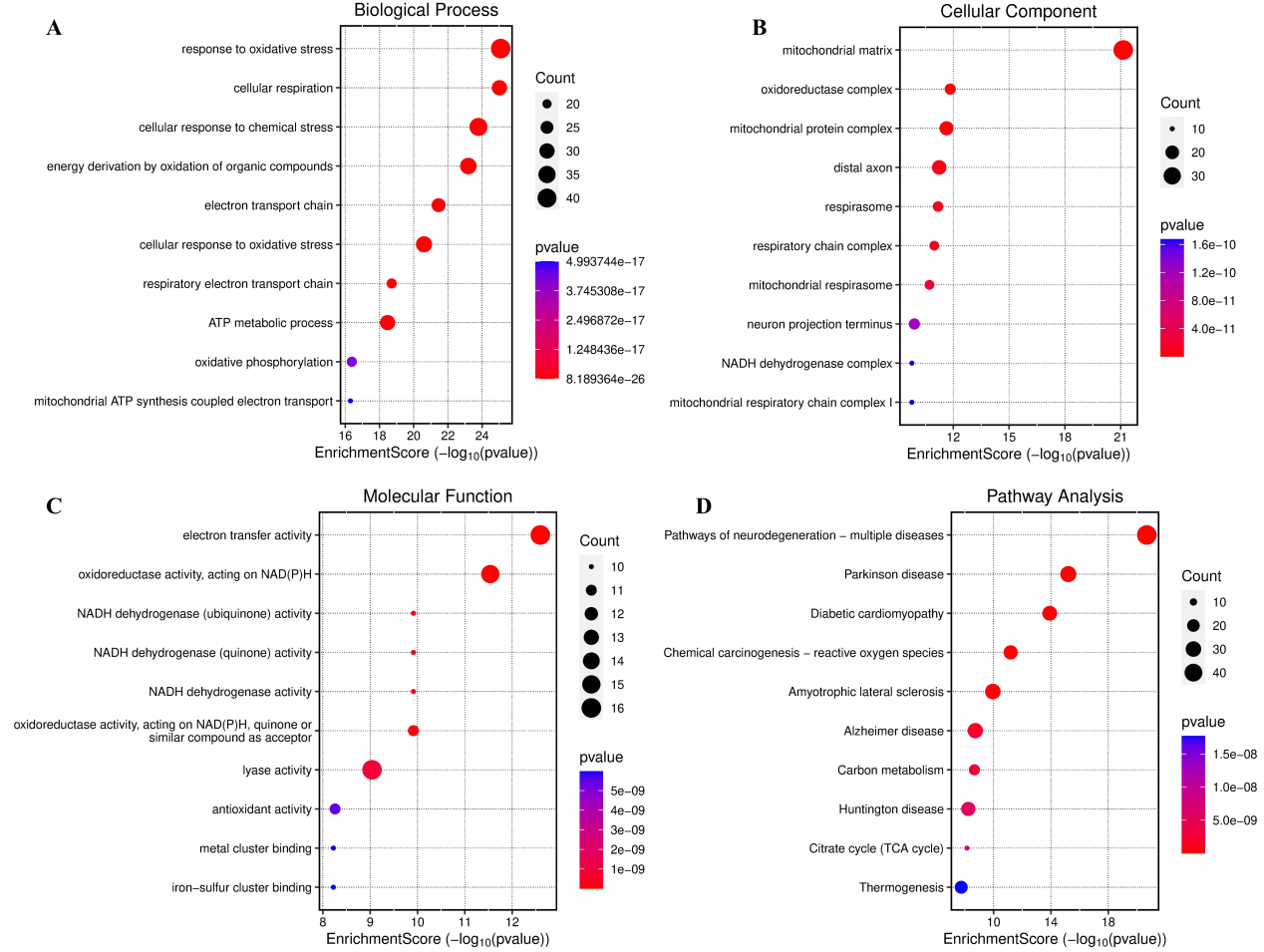


Figure S2 Biological function and KEGG enrichment analysis, including (A) GOBP, (B) GOCC, (C) GOMF, and (D) Pathway.


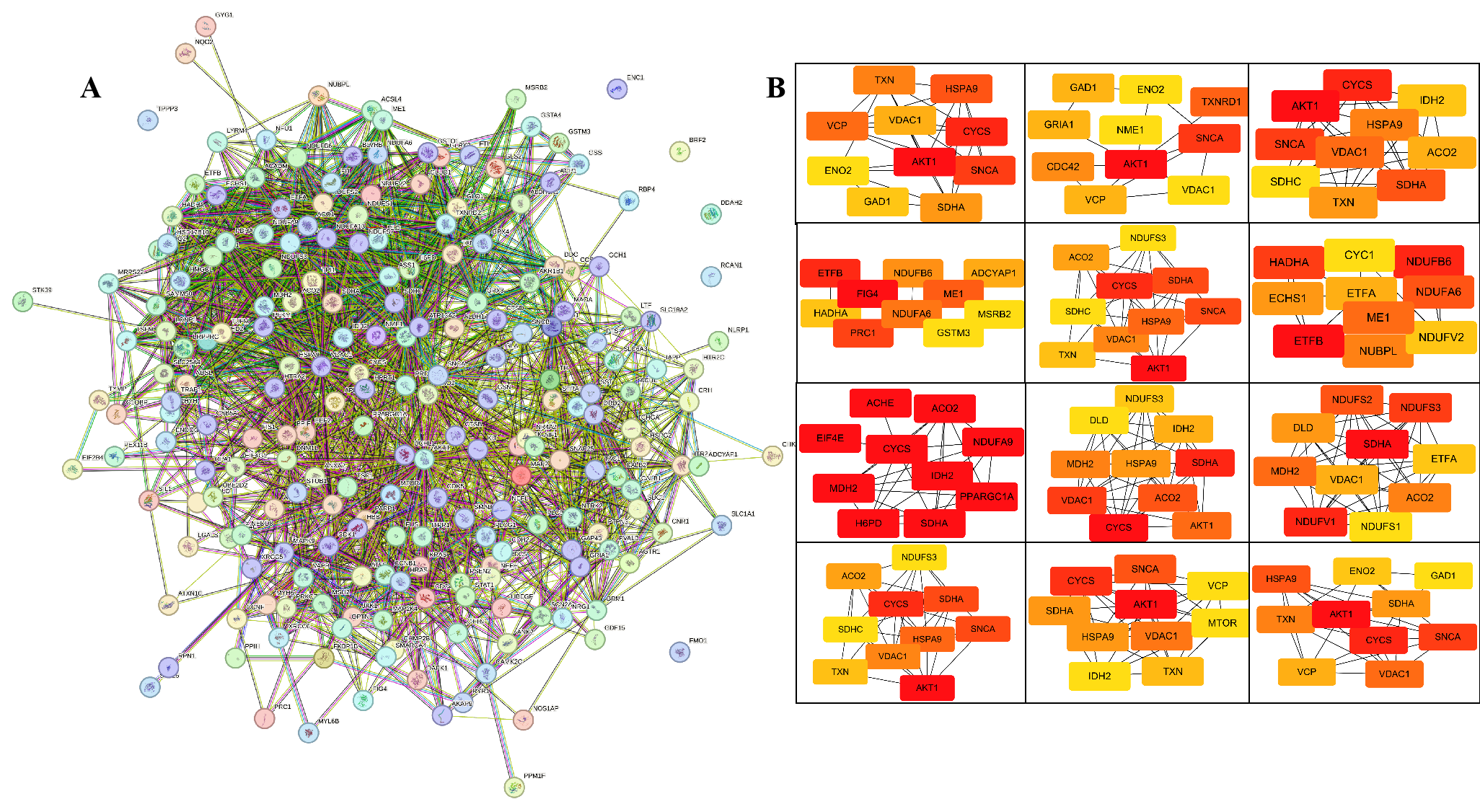


Figure S3 Hub gene analysis. (A) Protein-protein interaction network constituted with the 218 genes. (B) The 12 algorithms screen top 10 hub genes in Cytohubba, including Betweenness, BottleNeck, Closeness, ClusteringCoefficient, Degree, DMNC, EcCentricity, EPC, MCC, MNC, Radiality, and Stress.


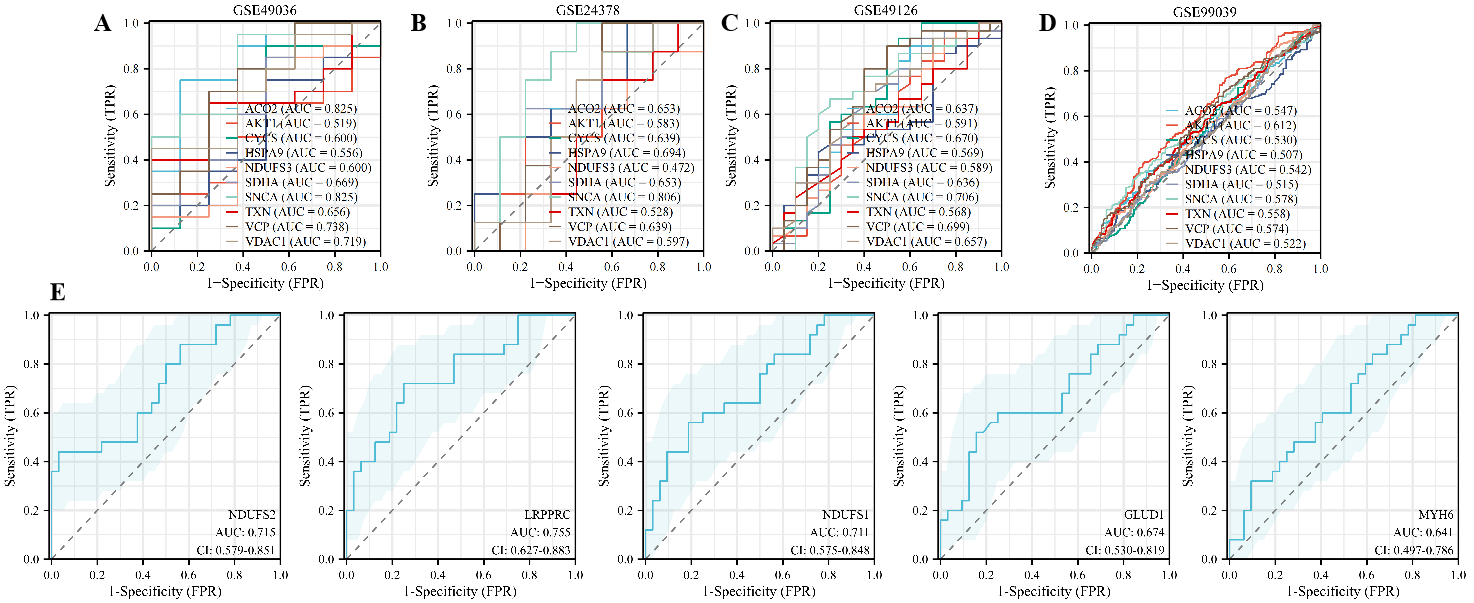


Figure S4 The diagnostic value of 10 hub genes includes (A) GSE49036 dataset, (B) GSE24378 dataset, (C) GSE49126 dataset, and (D) GSE99039 dataset. (E) Analysis of the diagnostic value of DED-ORGs.


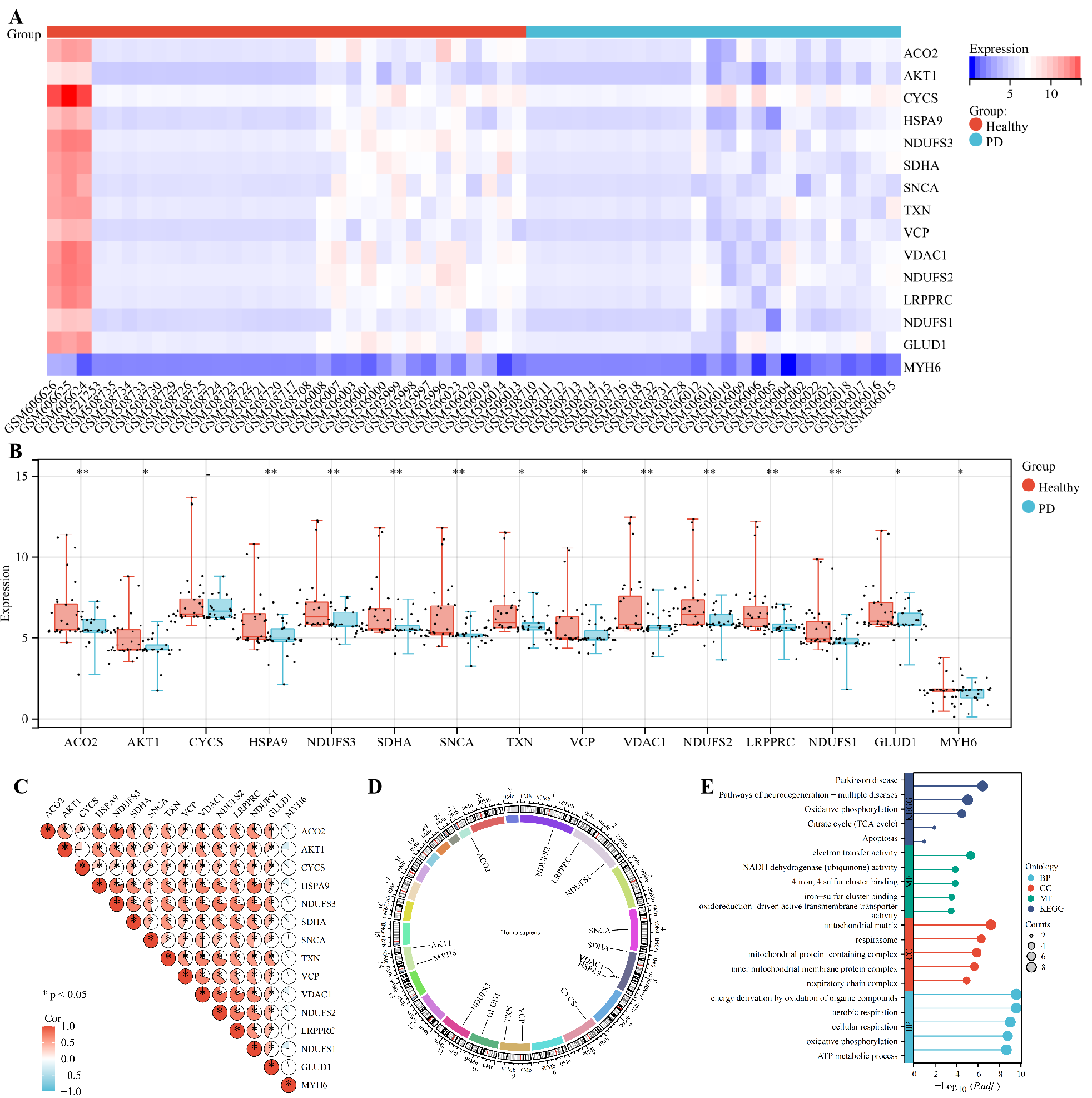


Figure S5 Hub genes and DED-ORGs analysis. (A) Heatmap of hub genes and DED-ORGs between the PD and healthy controls in the PD datasets. Red represents high expression, blue represents low expression. (B) Boxplot of hub genes and DED-ORGs between the PD and healthy controls in the PD datasets. (C) Spearman correlation among 15 genes in the PD datasets. Red represents positive correlation, blue represents negative correlation. (D) The genes are located on the chromosome. (E) Biological function and KEGG enrichment analysis.


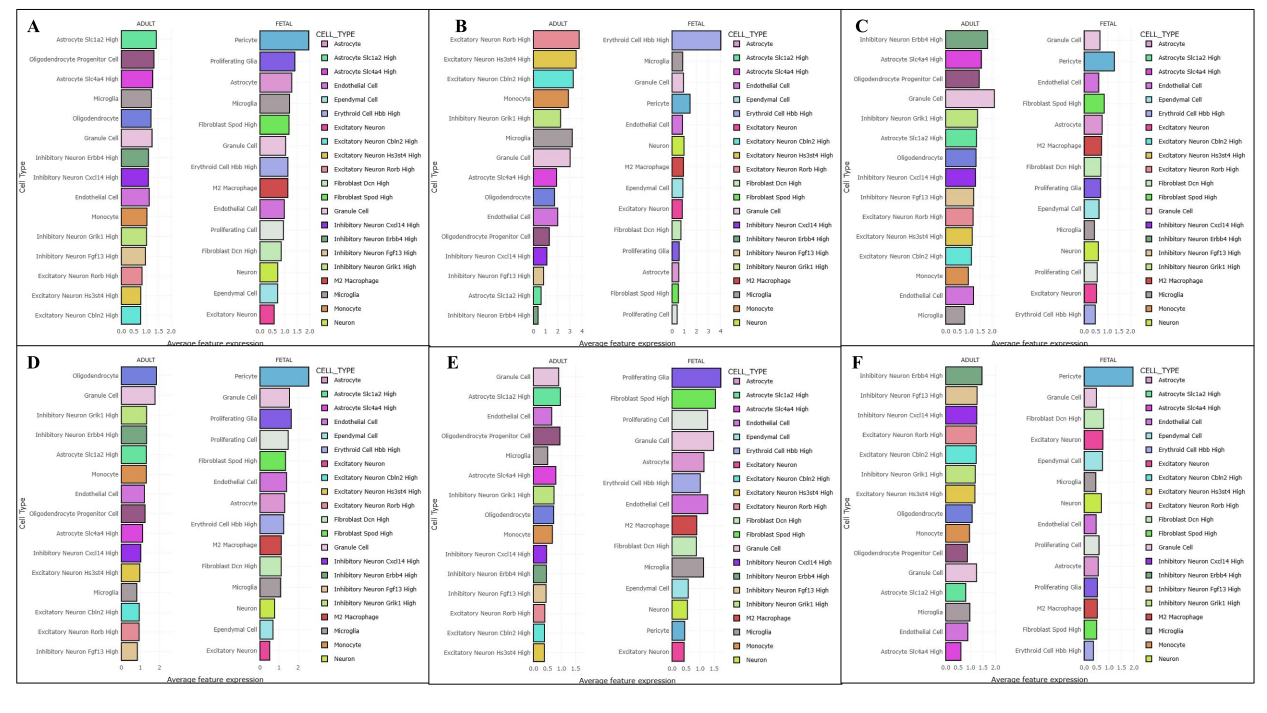


Figure S6 Single cell type analysis of (A) HSPA9, (B) SNCA, (C) NDUFS1, (D) NDUFS2, (E) NDUFS3, and (F) LRPPRC.


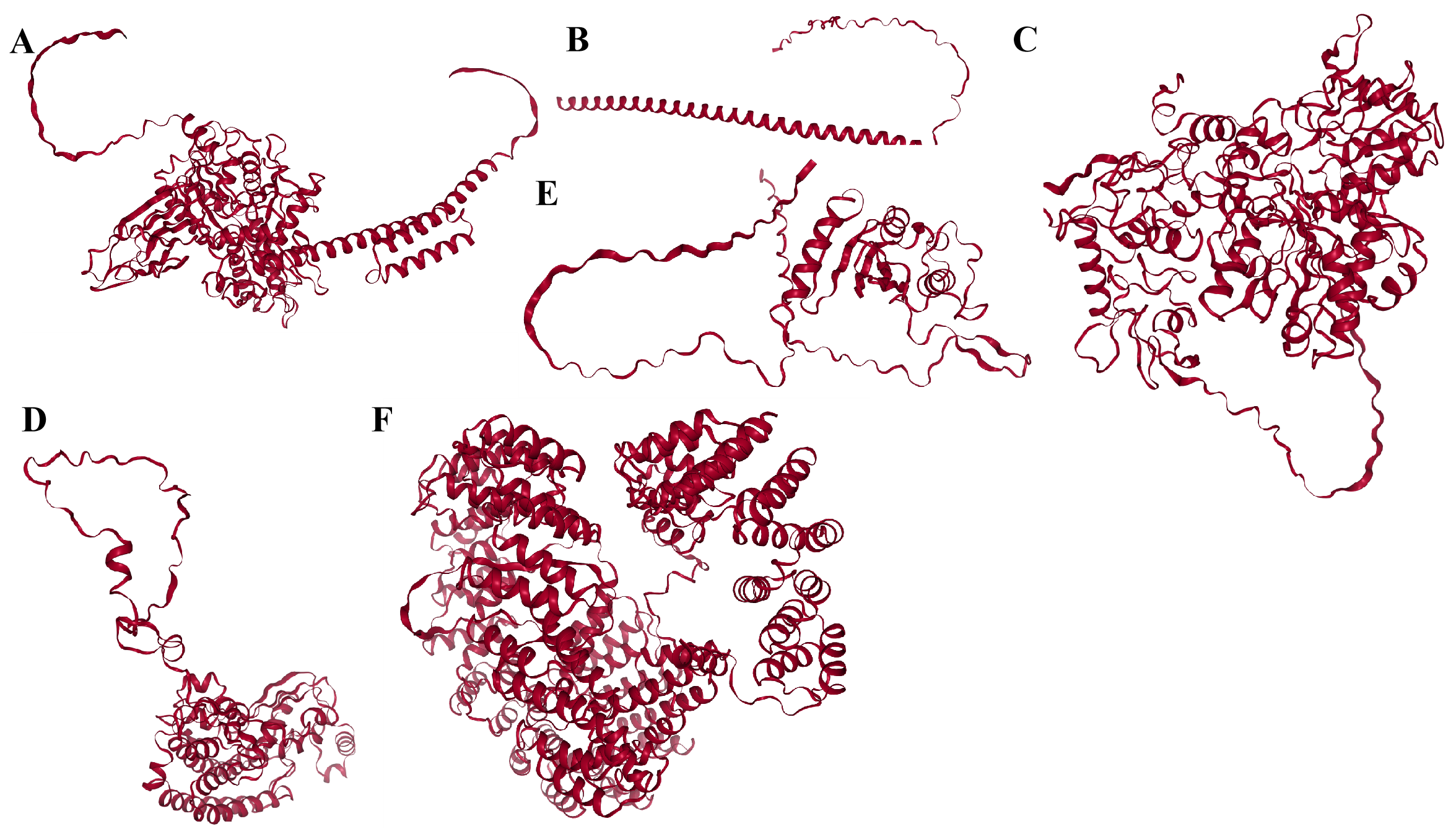


Figure S7 The structure of proteins, including, (A) HSPA9 (AF-P38646-F1), (B) SNCA (AF-P37840-F1), (C) NDUFS1 (AF-P28331-F1), (D) NDUFS2 (AF-O75306-F1), (E) NDUFS3 (AF-O75489-F1), and (F) LRPPRC (AF-P42704-F1).


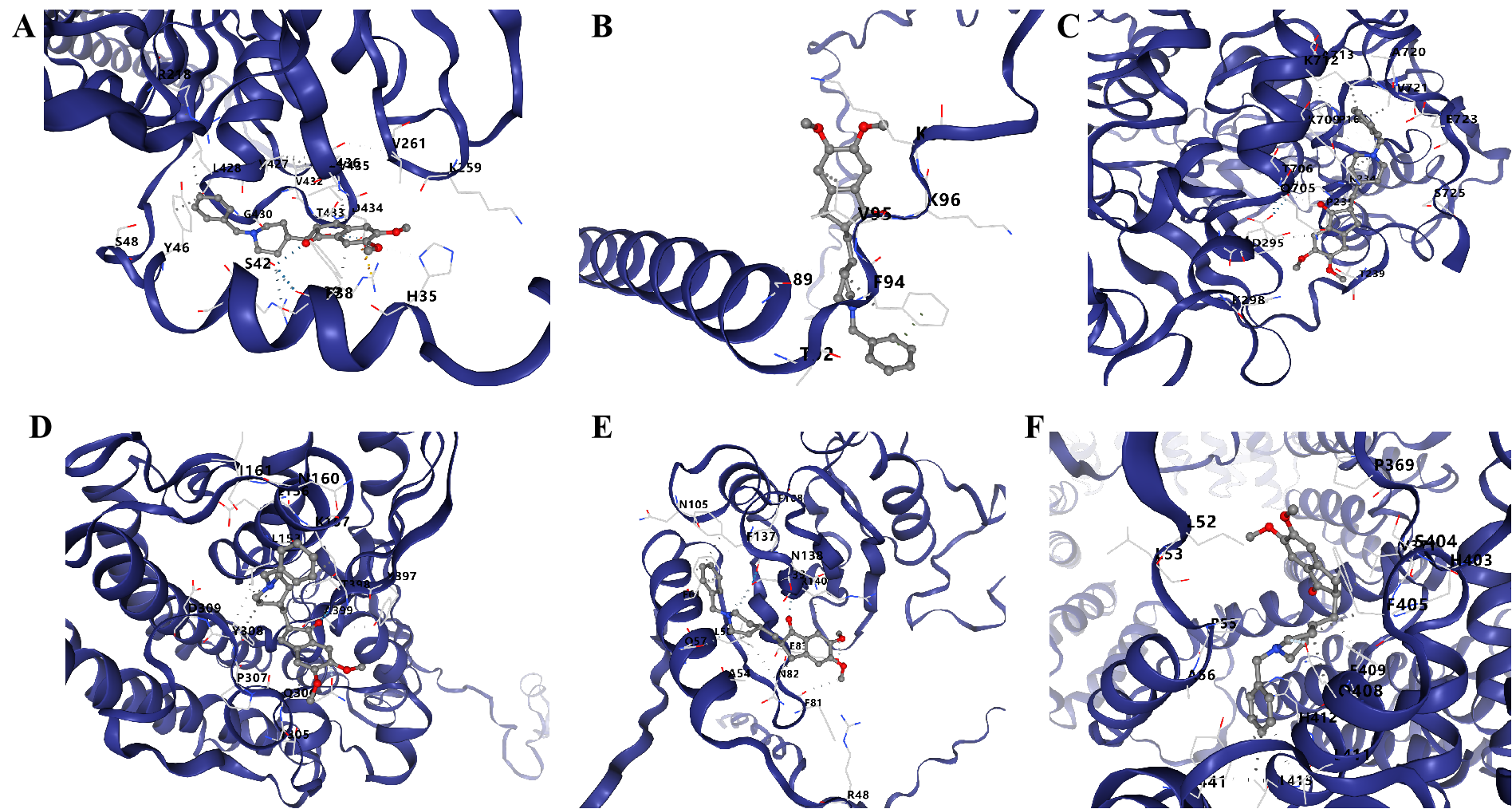


Figure S8 Molecular docking analysis docking sites, including (A) HSPA9 protein and Donepezil docking, (B) SNCA protein and Donepezil docking, (C) NDUFS1 protein and Donepezil docking, (D) NDUFS2 protein and Donepezil docking, (E) NDUFS3 protein and Donepezil docking, and (F) LRPPRC protein and Donepezil docking.
